# Enfortumab Vedotin With or Without Pembrolizumab in Metastatic Urothelial Carcinoma: A Systematic Review and Meta-Analysis

**DOI:** 10.1001/jamanetworkopen.2025.0250

**Published:** 2025-03-11

**Authors:** Shugo Yajima, Kohei Hirose, Hitoshi Masuda

**Affiliations:** 1 Department of Urology, National Cancer Center Hospital East, Chiba, Japan

## Abstract

**Importance:**

Metastatic urothelial carcinoma (mUC) presents a therapeutic challenge with poor outcome. Enfortumab vedotin has emerged as a promising treatment, necessitating a comprehensive evaluation of its effectiveness and safety.

**Objective:**

To synthesize the available evidence on enfortumab vedotin, both as monotherapy and in combination with pembrolizumab, as an mUC treatment for the purpose of guiding clinical decision-making and future research.

**Data Sources:**

Cochrane Library, MEDLINE (via PubMed), Google Scholar, and Web of Science were searched from database inception to August 31, 2024. Major conference abstracts from 2019 to 2024 were also included. Search strategy used a combination of Medical Subject Heading terms and free-text keywords related to mUC and enfortumab vedotin.

**Study Selection:**

Randomized clinical trials and prospective studies investigating enfortumab vedotin in adult patients with mUC were included. Eleven studies met the inclusion criteria.

**Data Extraction and Synthesis:**

Two independent reviewers extracted data and assessed study quality using the Cochrane Risk of Bias tool 2 and Risk of Bias in Non-Randomized Studies of Interventions tool. The meta-analysis used a random effects model, while a network meta-analysis was performed using a frequentist approach.

**Main Outcomes and Measures:**

Primary outcomes were disease control rate (DCR), objective response rate (ORR), and 1-year survival rate.

**Results:**

The 11 included studies (3 randomized clinical trials [27.3%] and 8 nonrandomized prospective studies [72.7%]) involved 2128 patients. Of these patients, 563 (26.5%) received enfortumab vedotin plus pembrolizumab, 814 (38.3%) received enfortumab vedotin without pembrolizumab, and 751 (35.3%) received chemotherapy. Enfortumab vedotin plus pembrolizumab was associated with a pooled DCR of 86% (95% CI, 83%-89%), ORR of 68% (95% CI, 64%-71%), and a 1-year survival rate of 79% (95% CI, 75%-82%). Enfortumab vedotin monotherapy had a pooled DCR of 73% (95% CI, 70%-76%), ORR of 43% (95% CI, 40%-47%), and a 1-year survival rate of 52% (95% CI, 48%-56%). Network meta-analysis revealed that enfortumab vedotin plus pembrolizumab significantly outperformed chemotherapy in ORR (odds ratio [OR], 3.47; 95% CI, 1.49-8.09; *P* = .004) and 1-year survival (OR, 2.32; 95% CI, 1.75-3.06; *P* < .001).

**Conclusions and Relevance:**

In this systematic review and meta-analysis, enfortumab vedotin plus pembrolizumab showed high response rates in first-line settings, while enfortumab vedotin monotherapy was associated with clinical benefit in later lines of therapy. These findings underscore the importance of personalized treatment approaches, and future research is warranted to refine enfortumab vedotin–based therapies for mUC management.

## Key Points

**Question:**

Is enfortumab vedotin, alone or combined with pembrolizumab, associated with disease control, objective response, and survival rates among patients with metastatic urothelial carcinoma?

**Findings:**

In this systematic review and meta-analysis of 11 studies involving 2128 patients, enfortumab vedotin plus pembrolizumab was associated with high objective response rate and high 1-year survival rate.

**Meaning:**

The findings suggest that enfortumab vedotin, especially combined with pembrolizumab, offers promising beneficial outcomes in metastatic urothelial carcinoma treatment; future research is warranted to refine enfortumab vedotin–based therapies for management of metastatic urothelial carcinoma.

## Teaser

This meta-analysis compares the disease control rate, objective response rate, 1-year survival rate, and adverse events associated with enfortumab vedotin–based therapy in adults with metastatic urothelial carcinoma.

## Introduction

Metastatic urothelial carcinoma (mUC) presents a global health challenge, with a poor 5-year survival rate typically less than 10% despite treatment advancements.^1,2^ The therapeutic landscape has evolved considerably, with platinum-based chemotherapy regimens traditionally forming the first-line treatment.^3^ However, their effectiveness is limited. The introduction of immune checkpoint inhibitors (ICIs) has shifted mUC management, demonstrating durable responses in some patients.^4^ Yet, overall response rates to ICIs remain suboptimal, with objective response rates (ORRs) of approximately 20%, highlighting the need for more effective treatments.^5,6,7^

Enfortumab vedotin, an antibody-drug conjugate targeting nectin-4, has emerged as a promising agent.^8^ Initially approved as monotherapy in the third-line setting based on the EV-201 (A Study of Enfortumab Vedotin for Patients With Locally Advanced or Metastatic Urothelial Bladder Cancer) trial, enfortumab vedotin has rapidly emerged as a cornerstone therapy. Its novel mechanism of action potentially overcomes resistance to conventional therapies.^9,10^ The combination of enfortumab vedotin and pembrolizumab has shown promising results in clinical trials.^11^

Given the evolving mUC treatment landscape, a comprehensive evaluation of enfortumab vedotin’s effectiveness and safety is imperative. This meta-analysis aimed to synthesize available evidence on enfortumab vedotin, both as monotherapy and in combination with pembrolizumab, as an mUC treatment for the purpose of guiding clinical decision-making and future research.

## Methods

We conducted a systematic review, meta-analysis, and network meta-analysis (NMA) and reported findings in accordance with the Preferred Reporting Items for Systematic Reviews and Meta-Analyses (PRISMA) reporting guideline.^12^ The protocol of this meta-analysis was preregistered in PROSPERO (identifier CRD42024582503).

### Search Strategy and Study Selection

A comprehensive literature search was conducted using 4 major databases: Cochrane Library, MEDLINE (via PubMed), Google Scholar, and Web of Science. The search strategy used a combination of Medical Subject Heading terms and free-text keywords related to mUC and enfortumab vedotin. The full search strategy for each database is provided in the eAppendix in Supplement 1. The literature search was conducted from database inception to August 31, 2024.

**Figure 1.**
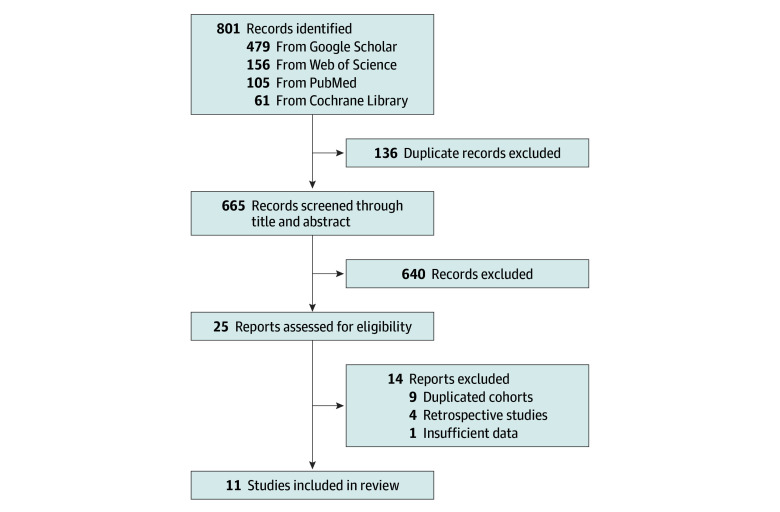
Flow Diagram of Literature Search and Study Selection From 801 initial records, 11 studies were ultimately included after removing duplicates, screening abstracts, and assessing full-text eligibility.

A PRISMA flow diagram was created to illustrate the study selection process (Figure 1). We included randomized clinical trials (RCTs) and prospective studies investigating the beneficial outcomes and safety of enfortumab vedotin, either as monotherapy or in combination with pembrolizumab, in adult patients with mUC. Studies were eligible for inclusion regardless of language or publication status. To capture the most recent data, we also included abstracts from major conferences—specifically the European Society for Medical Oncology congress and the American Society of Clinical Oncology annual meeting—for the past 5 years.

Exclusion criteria were applied to ensure the quality and relevance of the included studies. We excluded studies explicitly labeled as *real world data* or *retrospective* in their titles. Additionally, review articles, commentaries, editorials, and preclinical studies were not considered for inclusion. Case reports and case series were also excluded due to their limited generalizability.

### Data Extraction and Quality Assessment

Two of us (S.Y. and K.H.) extracted data from the included studies. Extracted information included study characteristics, patient demographics, intervention details, outcome measures, and follow-up duration. For duplicate cohorts, we selected the study with the larger sample size or more recent publication. Any discrepancies in data extraction were resolved through discussion, with another coauthor (H.M.) serving as the arbiter when necessary.

The methodological quality of the included studies was assessed using the Cochrane Risk of Bias tool 2 (RoB 2)^13^ for RCTs and the Risk of Bias in Non-Randomized Studies of Interventions (ROBINS-I) tool^14^ for nonrandomized prospective studies. These assessments were conducted independently by 2 of us (S.Y. and K.H.), with disagreements resolved through consensus or consultation with our coauthor (H.M.). Additionally, for the major outcomes of the NMA, we evaluated the reliability of the results using the Confidence in Network Meta-Analysis (CINeMA) web application^15^ to ensure a comprehensive assessment of the evidence quality. In the CINeMA analysis, we defined the clinically important effect size for odds ratios (ORs) as 2, meaning that an OR of 2 or greater was considered clinically significant.

### Outcomes of Interest

In the systematic review and meta-analysis, we collected data on various outcomes from the included studies. The primary outcomes were disease control rate (DCR), ORR, and 1-year survival rate. DCR was defined as the proportion of patients achieving complete response, partial response, or stable disease, while ORR was the proportion of patients achieving complete or partial response. The 1-year survival rate represented the proportion of patients alive at the 1-year time point. When not explicitly stated, 1-year survival rates were extracted from Kaplan-Meier curves or survival data tables.

The secondary outcomes focused on safety profiles, specifically the incidence of all-grade and high-grade (grade 3 or higher) treatment-related adverse events (AEs). We also collected data on overall survival (OS) and progression-free survival where available, extracting hazard ratios (HRs) with 95% CIs when reported. Due to limited reporting, OS and progression-free survival were not included in the meta-analysis but were presented descriptively.

We calculated rates using the appropriate denominator for each outcome, accounting for potential differences in evaluable populations within individual studies. This approach ensured the most accurate representation of the data. In cases of unclear or missing information, we contacted study authors for clarification. When clarification was not possible, we documented the missing data and excluded the data from relevant analyses.

### Statistical Analysis

All statistical analyses were conducted using R, version 4.3.1 (R Project for Statistical Computing). Statistical tests were 2-sided, with significance set at *P* < .05. Heterogeneity was assessed using the *I*^2^ statistic and Cochran *Q* test, with *I*^2^ values of 25%, 50%, and 75% indicating low, moderate, and high heterogeneity, respectively.

For the meta-analysis, we used random-effects models to account for the anticipated between-study heterogeneity. When heterogeneity was significant (*P* < .05 in Cochran *Q* test), we investigated its cause. Meta-analyses were performed using pooled proportions for DCR, ORR, 1-year survival rate, and AE incidence. Sensitivity analyses were planned based on treatment regimen and therapy line.

For the NMA comparing enfortumab vedotin monotherapy, enfortumab vedotin plus pembrolizumab, and chemotherapy, we used a frequentist, contrast-based approach with the netmeta package in R.^16^ Treatment effects and SEs were calculated from the outcomes and used to estimate ORs and 95% CIs. The NMA was performed using the netmeta function in R. League tables and surface under the cumulative ranking (SUCRA) curves were produced to rank treatment effectiveness.

## Results

### Study Characteristics

The initial literature search identified 801 records. After removing 136 duplicate records, 665 records remained for screening of titles and abstracts (Figure 1). Following this screening, 640 articles were excluded, leaving 25 articles or abstracts for full-text review.^2,9,11,17,18,19,20,21,22,23,24,25,26,27,28,29,30,31,32,33,34,35,36,37,38^ From these 25 works, 14 were excluded based on the inclusion criteria (4 retrospective studies,^2,17,18,19^ 9 duplicated cohorts,^9,20,21,22,23,24,25,26,27^ and 1 with insufficient data^28^). We ultimately included 11 studies comprising 2128 patients (563 [26.5%] received enfortumab vedotin plus pembrolizumab, 814 [38.3%] received enfortumab vedotin monotherapy, and 751 [35.3%] received chemotherapy).^11,29,30,31,32,33,34,35,36,37,38^

Of the 11 included studies, 3 (27.3%) were RCTs^11,29,30^ and 8 (72.7%) were nonrandomized prospective studies.^31,32,33,34,35,36,37,38^ Regarding interventions, 7 studies^29,31,32,33,35,36,38^ administered enfortumab vedotin monotherapy, 3 studies^11,30,37^ administered enfortumab vedotin plus pembrolizumab, and 1 study^34^ administered enfortumab vedotin plus sacituzumab govitecan. The Table shows the characteristics and outcomes of each of the 11 studies, which were published between 2019 and 2024. Detailed background, treatment schedules, and patient conditions for each study are presented in eTable 1 in Supplement 1, which also provides context, including patient demographics, disease characteristics, treatment details, prior therapy, and Eastern Cooperative Oncology Group Performance Status score.

**Table.** Characteristics and Outcomes of the Clinical Trials Included in This Meta-Analysis

Study name; source; design	Patients, No./total No. (%)		Arm		Responses or disease control/total evaluated, No. (%)		1-y Survival rate, % (95% CI)	PFS		OS		TRAEs		
	Treatment	Control	Treatment	Control	ORR	DCR		Median (95% CI), mo	HR (95% CI)	Median (95% CI), mo	HR (95% CI)	No./total No. (%)		Death, No.
												Overall	High-grade	
EV-302; Powles et al,^11^ 2024; RCT	442/886 (49.9)^a^	444/886 (50.1)^b^	Enfortumab vedotin plus pembrolizumab	Chemotherapy^c^	296/437 (67.7)	378/437 (86.5)	78.2 (73.9-81.9)	12.5 (10.4-16.6)	0.45 (0.38-0.54)	31.5 (25.4-NR)	0.47 (0.38-0.58)	429/440 (97.0)	246/440 (55.9)	4
EV-301; Rosenberg et al,^29^ 2023; RCT^d^	301/608 (49.5)^e^	307/608 (50.5)^f^	Enfortumab vedotin	Chemotherapy^g^	119/288 (41.3)	207/288 (71.9)	51.5 (44.6-58.0)	5.55 (5.32-5.82)	0.63 (0.53-0.76)	12.88 (10.58-15.21)	0.70 (0.58-0.85)	278/296 (93.9)	152/296 (52.4)	2
EV-103 Cohort K; O’Donnell et al,^30^ 2023; RCT	76/149 (51.0)	73/149 (49.0)	Enfortumab vedotin plus pembrolizumab	Enfortumab vedotin	49/76 (64.5)	66/76 (86.8)	80.7 (69.4-88.1)	NA	NA	22.3 (19.09-NR)	NA	NA	48/76 (63.2)	3
EV-101; Rosenberg et al,^31^ 2020; phase 1 dose escalation/dose expansion study	155/155 (100)^h^	NA	Enfortumab vedotin	NA	48/112 (42.9)	80/112 (71.4)	51.8 (NA)	5.4 (5.1-6.3)	NA	12.3 (9.3-15.3)	NA	145/155 (93.5)	53/155 (34.2)	4
EV-201 Cohort 1; Rosenberg et al,^32^ 2019; phase 2, single-arm, multicenter study	125/125 (100)	NA	Enfortumab vedotin	NA	55/125 (44.0)	90/125 (71.9)	51.2 (42.1-60.3)	5.8 (4.9-7.5)	NA	11.7 (9.1-NR)	NA	117/125 (93.6)	68/125 (54.4)	0
EV-201 Cohort 2; Yu et al,^33^ 2021; phase 2, single-arm, multicenter study	89/89 (100)	NA	Enfortumab vedotin	NA	46/89 (51.7)	73/89 (82.0)	52.9 (41.2-64.6)	5.8 (5.0-8.3)	NA	14.7 (10.5-18.2)	NA	86/89 (96.6)	49/89 (55.1)	4
DAD; McGregor et al,^34^ 2024; phase 1	23/23 (100)	NA	Enfortumab vedotin plus sacituzumab govitecan	NA	16/23 (69.6)	19/23 (82.6)	86 (61-95)	NA	NA	NA	NA	NA	18/23 (78.3)	1
NA; Taoka et al,^35^ 2024; prospective, multicenter, cohort study	34/34 (100)	NA	Enfortumab vedotin	NA	18/34 (52.9)	25/34 (73.5)	54.5 (NA)	6.95 (NA)	NA	13.5 (NA)	NA	26/34 (76.5)	NA	0
EV-102; Takahashi et al,^36^ 2020; phase 1	17/17 (100)	NA	Enfortumab vedotin	NA	6/17 (35.3)	13/17 (76.5)	NA	8.1 (3.5-NR)	NA	NA	NA	15/17 (88.2)	NA	0
EV-103 Cohort A; Gupta et al,^37^ 2023; phase 1b/2	45/45 (100)	NA	Enfortumab vedotin plus pembrolizumab	NA	33/45 (73.3)	38/45 (84.4)	83.4 (68.3-91.7)	12.7 (6.1-NR)	NA	26.1 (15.5-NR)	NA	NA	NA	NA
EV-203; Li et al,^38^ 2023; phase 2	40/40 (100)	NA	Enfortumab vedotin	NA	15/40 (37.5)	29/40 (72.5)	NA	4.67 (NA)	NA	NA	NA	NA	NA	NA

Abbreviations: DCR, disease control rate; HR, hazard ratio; NA, not applicable; NR, not reached; ORR, objective response rate; OS, overall survival; PFS, progression-free survival; RCT, randomized clinical trial; TRAEs, treatment-related adverse events.

^a^ A total of 440 patients were eligible for the assessment of adverse events (AEs), while 437 patients were eligible for the evaluation of treatment effectiveness.

^b^ A total of 433 patients were eligible for the assessment of AEs, while 441 patients were eligible for the evaluation of treatment efficacy.

^c^ Gemcitabine and either cisplatin or carboplatin.

^d^ The information also included data from another report of the same cohort.^9^

^e^ A total of 296 patients were eligible for the assessment of AEs, while 288 patients were eligible for the evaluation of ORR and DCR.

^f^ A total of 291 patients were eligible for the assessment of AEs.

^g^ Docetaxel, paclitaxel, or vinflunine.

^h^ A total of 112 patients were eligible for the evaluation of treatment effectiveness.

### Study Quality 

The RoB 2 assessment revealed that 2 of the 3 RCTs^11,29^ had a low risk of bias across all domains (eFigure 1 in Supplement 1). The RCT by O’Donnell et al^30^ showed some concerns in the domain of deviations from intended interventions, while maintaining low risk in all other domains. These concerns emerged because, although the study reported treatment discontinuations and modifications, it did not specify whether an intention-to-treat analysis was conducted, nor did it provide clear information on crossover treatment. Overall, the quality of the RCTs was high, with only minor concerns in 1 study.

For nonrandomized studies, the ROBINS-I assessment indicated varying levels of bias (eFigure 1 in Supplement 1). Two studies^32,33^ demonstrated low risk of bias across all domains. Conversely, 2 studies^37,38^ were only available as abstracts, precluding a comprehensive bias assessment due to insufficient information. However, 4 studies^31,34,35,36^ were judged to have moderate overall risk of bias, primarily based on concerns in the domain of bias due to confounding. These studies showed low risk of bias in all other domains.

### Meta-Analysis Results

Primary outcomes are presented in eFigure 2 in Supplement 1. The pooled DCR was 79% (95% CI, 74%-83%), with moderate heterogeneity (*I*^2^ = 73%; *P* < .001). The pooled ORR was 53% (95% CI, 45%-61%), demonstrating high heterogeneity (*I*^2^ = 88%; *P* < .001). The pooled 1-year survival rate was 67% (95% CI, 56%-76%), also demonstrating high heterogeneity (*I*^2^ = 92%; *P* < .001). Regarding secondary outcomes, the pooled incidence of all-grade AEs was 94% (95% CI, 90%-96%), with high heterogeneity (*I*^2^ = 75%; *P* < .001), and the pooled incidence of high-grade AEs was 55% (95% CI, 47%-62%), also exhibiting high heterogeneity (*I*^2^ = 78%; *P* < .001) (eFigure 3 in Supplement 1). Results for specific AEs are provided in eFigure 4 in Supplement 1.

The observed heterogeneity was attributed to the inclusion of studies with both enfortumab vedotin monotherapy and enfortumab vedotin combination therapies. Consequently, we conducted sensitivity analyses separately for enfortumab vedotin monotherapy and enfortumab vedotin plus pembrolizumab. Initially, we intended to perform additional sensitivity analyses based on first-line vs second-line or later treatments. However, all enfortumab vedotin plus pembrolizumab studies (n = 3)^11,30,37^ were first-line treatments, while all enfortumab vedotin monotherapy studies (n = 7)^29,31,32,33,35,36,38^ were second-line or later treatments, precluding this analysis.

#### Enfortumab Vedotin Monotherapy

**Figure 2.**
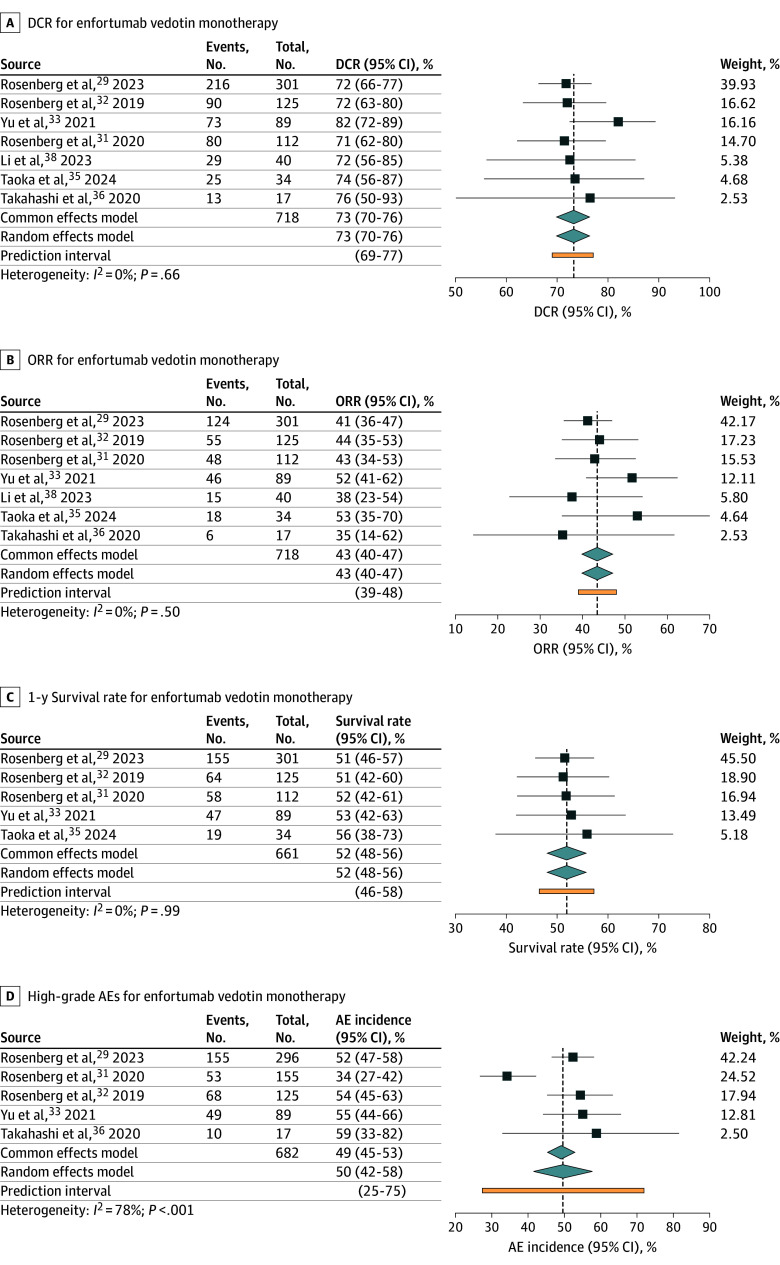
Disease Control Rate (DCR), Objective Response Rate (ORR), 1-Year Survival Rate, and High-Grade Adverse Events (AEs) for Enfortumab Vedotin Monotherapy in Metastatic Urothelial Carcinoma Plots show individual study results and pooled estimates with 95% CIs. Squares represent individual study effect sizes, and diamonds represent the pooled or overall effect estimates from meta-analysis, with the width of the diamond showing the 95% CI.

Results of primary and secondary outcomes in enfortumab vedotin monotherapy studies^29,31,32,33,35,36,38^ are presented in Figure 2. The pooled DCR was 73% (95% CI, 70%-76%) with no heterogeneity (*I*^2^ = 0%; *P* = .66). The pooled ORR was 43% (95% CI, 40%-47%; *I*^2^ = 0%; *P* = .50), and the pooled 1-year survival rate was 52% (95% CI, 48%-56%; *I*^2^ = 0%; *P* = .99), both demonstrating no heterogeneity. For secondary outcomes, the pooled incidence of all-grade AEs was 93% (95% CI, 88%-95%), with moderate heterogeneity (*I*^2^ = 66%; *P* = .001), and the pooled incidence for high-grade AEs was 50% (95% CI, 42%-58%), which had high heterogeneity (*I*^2^ = 78%; *P* < .001). This heterogeneity was attributed to differences in patient populations across studies (eTable 1 in Supplement 1). Results for specific complications and all-grade AEs (overall) are provided in eFigure 5 in Supplement 1.

#### Enfortumab Vedotin Plus Pembrolizumab

**Figure 3.**
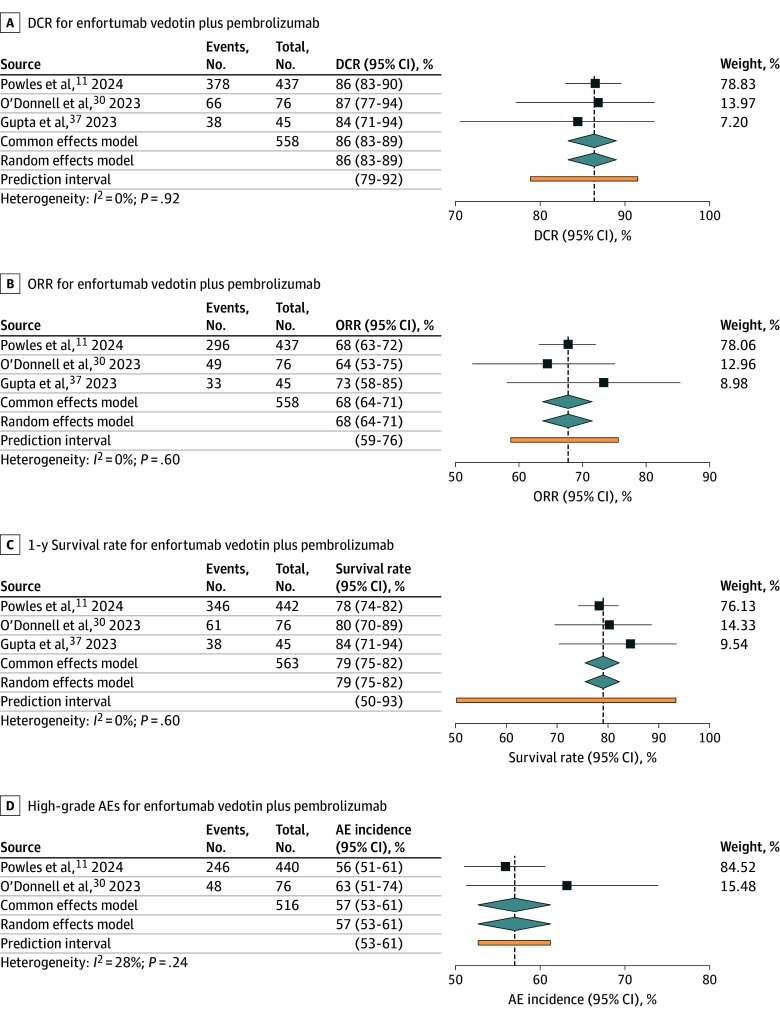
Disease Control Rate (DCR), Objective Response Rate (ORR), 1-Year Survival Rate, and High-Grade Adverse Events (AEs) for Enfortumab Vedotin Plus Pembrolizumab in Metastatic Urothelial Carcinoma Plots show individual study results and pooled estimates with 95% CIs. Squares represent individual study effect sizes, and diamonds represent the pooled or overall effect estimates from meta-analysis, with the width of the diamond showing the 95% CI.

Results of primary and secondary outcomes in enfortumab vedotin plus pembrolizumab studies^11,30,37^ are presented in Figure 3. The pooled DCR was 86% (95% CI, 83%-89%) with no heterogeneity (*I*^2^ = 0%; *P* = .92). The pooled ORR was 68% (95% CI, 64%-71%; *I*^2^ = 0%; *P* = .60), and the pooled 1-year survival rate was 79% (95% CI, 75%-82%; *I*^2^ = 0%; *P* = .60), both demonstrating no heterogeneity. Regarding secondary outcomes, the incidence of all-grade AEs could be evaluated in only 1 study.^11^ The pooled incidence of high-grade AEs was 57% (95% CI, 53%-61%), with low heterogeneity (*I*^2^ = 28%; *P* = .24). Results for specific complications and all-grade AEs (overall) are provided in eFigure 6 in Supplement 1.

### Network Meta-Analysis Results

The NMA compared the effectiveness and safety of enfortumab vedotin, enfortumab vedotin plus pembrolizumab, and chemotherapy in the treatment of metastatic urothelial carcinoma. This analysis was based on 3 key RCTs,^11,29,30^ enabling a comprehensive comparison across multiple outcomes. The network of evidence for this comparison is presented in eFigure 7 in Supplement 1.

#### Comparative Effectiveness

**Figure 4.**
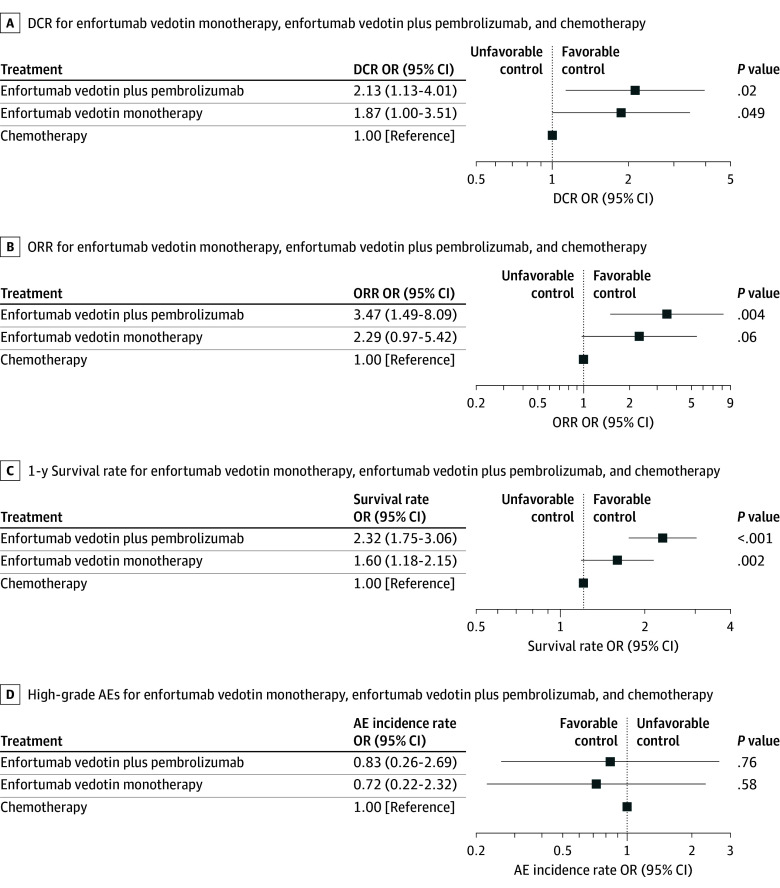
Results of Network Meta-Analysis Comparing Enfortumab Vedotin Monotherapy, Enfortumab Vedotin Plus Pembrolizumab, and Chemotherapy in Metastatic Urothelial Carcinoma Plots show odds ratio (ORs) with 95% CIs. AE indicates adverse event; DCR, disease control rate; ORR, objective response rate.

For DCR, both enfortumab vedotin plus pembrolizumab (OR, 2.13; 95% CI, 1.13-4.01; *P* = .02) and enfortumab vedotin monotherapy (OR, 1.87; 95% CI, 1.00-3.51; *P* = .049) were associated with higher response rates compared with chemotherapy (Figure 4A). Regarding ORR, enfortumab vedotin plus pembrolizumab was associated with higher rates compared with chemotherapy (OR, 3.47; 95% CI, 1.49-8.09; *P* = .004), whereas the comparison between enfortumab vedotin monotherapy and chemotherapy for ORR did not reach statistical significance (OR, 2.29; 95% CI, 0.97-5.42; *P* = .06) (Figure 4B). The 1-year survival rate analysis revealed that enfortumab vedotin plus pembrolizumab was associated with higher response rates compared with chemotherapy (OR, 2.32; 95% CI, 1.75-3.06; *P* < .001). Enfortumab vedotin monotherapy also showed a significant survival rate increase over chemotherapy (OR, 1.60; 95% CI, 1.18-2.15; *P* = .002) (Figure 4C). The certainty of evidence for these comparisons was assessed using the CINeMA approach (eTable 2 in Supplement 1).

Based on the SUCRA analysis, enfortumab vedotin plus pembrolizumab consistently had the highest probability of providing the greatest benefit across all key beneficial outcomes, as illustrated in eFigure 8 in Supplement 1. This combination therapy ranked first for DCR (0.81), ORR (0.91), and 1-year survival rate (0.99).

#### Comparative Safety 

The NMA evaluated the safety profiles of enfortumab vedotin monotherapy, enfortumab vedotin plus pembrolizumab, and chemotherapy by comparing AEs. Although we intended to analyze both all-grade and high-grade (≥grade 3) AEs, insufficient data precluded a comprehensive analysis of all-grade AEs across the 3 treatment arms. Therefore, the primary safety analysis focused on high-grade AEs. Figure 4D presents the results for high-grade AEs, showing that while enfortumab vedotin monotherapy (OR, 0.72; 95% CI, 0.22-2.32; *P* = .58) and enfortumab vedotin plus pembrolizumab (OR, 0.83; 95% CI, 0.26-2.69; *P* = .76) had numerically lower odds of high-grade AEs compared with chemotherapy, these differences were not statistically significant.

To provide a more comprehensive assessment of the safety profiles, we conducted in-depth analyses of common AEs associated with both chemotherapy and targeted therapies. Specifically, we examined high-grade AE occurrences of anemia, diarrhea, fatigue, neutropenia, pruritus, and peripheral neuropathy. Results for these specific high-grade AEs are presented in eFigure 9 in Supplement 1. Detailed analysis of specific adverse events revealed distinct safety profiles among treatments. For hematologic toxic effects, eFigure 11 in Supplement 1 shows that all-grade anemia was less frequent with EV plus pembrolizumab compared with chemotherapy (OR, 0.20; 95% CI, 0.05-0.84; *P* = .03). EV monotherapy was also associated with lower odds of anemia, although this difference was not statistically significant (OR, 0.29; 95% CI, 0.07-1.20; *P* = .09). Although not statistically significant, both EV-based regimens for neutropenia were associated with numerically lower odds (eFigure 11 in Supplement 1). In contrast, peripheral neuropathy analysis indicated higher frequency with EV plus pembrolizumab compared with chemotherapy (OR, 6.13; 95% CI, 1.97-19.10; *P* = .002). The SUCRA rankings for peripheral neuropathy were consistent with these findings, with chemotherapy ranking highest (0.77), followed by EV plus pembrolizumab (0.38) and EV monotherapy (0.35) (eFigure 10 in Supplement 1).

## Discussion

The systematic review, meta-analysis, and NMA conducted for this study provide a comprehensive evaluation of enfortumab vedotin in the treatment of mUC. The findings demonstrate that enfortumab vedotin, both as monotherapy and in combination with pembrolizumab, offers substantial clinical benefits compared with conventional chemotherapy.

Enfortumab vedotin plus pembrolizumab had an ORR of 68% and DCR of 86%, surpassing historical benchmarks for mUC treatments. These results represent a substantial leap forward in the management of this disease. Similarly, enfortumab vedotin monotherapy was associated with higher response rates compared with chemotherapy, particularly in later lines of treatment. Historically, overall response rates to ICIs have been suboptimal, with ORRs of approximately 20% in both first-line and second-line settings for mUC.^5,6,7^ Platinum-based chemotherapy has traditionally demonstrated ORRs of 40% to 50% in first-line treatment of mUC, with a median OS of 12 to 15 months across key studies.^39^ The superior benefits of enfortumab vedotin plus pembrolizumab, with a median (IQR) OS of 31.5 months (95% CI, 25.4 to not reached) in the first-line setting (EV-302 [Enfortumab Vedotin and Pembrolizumab vs Chemotherapy Alone in Untreated Locally Advanced or Metastatic Urothelial Cancer] trial),^11^ represent a substantial improvement over these historical chemotherapy outcomes. Similarly, while historical second-line chemotherapy studies have reported modest ORRs of 8.6% with vinflunine^40^ and 13.9% with docetaxel,^41^ the 43% ORR achieved with enfortumab vedotin monotherapy in the current study represents a clinically meaningful benefit for patients with limited therapeutic options.

The safety analysis revealed distinct AE patterns between enfortumab vedotin–based therapies and chemotherapy. While the overall incidence of high-grade AEs was comparable between treatment groups, specific toxic effect patterns showed notable differences. Enfortumab vedotin–based treatments were associated with lower rates of anemia and neutropenia but higher rates of peripheral neuropathy. NMA demonstrated an association between enfortumab vedotin–based regimens and peripheral neuropathy. The mechanism underlying this observation may be associated with microtubule disruption from the monomethyl auristatin E payload, which differs from traditional platinum-based chemotherapy-induced peripheral neuropathy through its role in axonal transport.^42^

Enfortumab vedotin–based regimens were also associated with increased rates of skin-related AEs, particularly rash and pruritus. This observation aligns with the known expression of nectin-4, the target of enfortumab vedotin, in epidermal keratinocytes and skin appendages.^43^ In the pivotal EV-302 trial, additional notable AEs included ocular disorders (21.0% vs 2.8% with chemotherapy) and hyperglycemia (13.0% vs 0.7% with chemotherapy).^11^ While these AEs were not consistently reported across all studies in the present meta-analysis, their observation in the large phase 3 trial warrants examination in future studies. This distinct toxic effect profile highlights the importance of tailored AE management in clinical practice.

These findings have implications for how to approach patient stratification in mUC. While cisplatin eligibility has traditionally been the primary factor guiding first-line treatment selection, the superior outcomes of enfortumab vedotin plus pembrolizumab across all populations challenge this paradigm.^44^ This shift suggests a need to develop new molecular and clinical criteria that better reflect treatment selection in the current therapeutic landscape.

### Limitations

Several study limitations warrant consideration in the interpretation of this report. The inability to calculate exposure-adjusted incidence rates constrained our capacity to fully account for disparities in treatment duration. The small number of RCTs (n = 3) in the NMA may affect the robustness of these comparative analyses. A critical consideration is the inherent disparity in treatment settings between enfortumab vedotin monotherapy and enfortumab vedotin plus pembrolizumab studies, which may lead to an overestimation of the combination therapy’s effectiveness. Furthermore, while the preponderance of RCTs mitigated publication bias concerns, the absence of a formal assessment may have overlooked potential sources of bias.

## Conclusions

In this meta-analysis of 11 studies, enfortumab vedotin–based therapy was associated with favorable outcomes in mUC treatment settings. Enfortumab vedotin plus pembrolizumab was associated with higher response rates in the first-line setting, while enfortumab vedotin monotherapy was associated with clinical benefit in later lines. The distinct profiles of these regimens underscore the importance of personalized treatment approaches. Ongoing research is crucial to further refine enfortumab vedotin–based therapies and improve outcomes for mUC patients.

## Notes

Supplement 1. **eAppendix.** Search Strategy for Meta-analysis **eTable 1.** Clinical Parameters and Results of the Trials Incorporated in This Analysis **eFigure 1.** The RoB2 Assessment (A) and the ROBINS-I Assessment (B) of the Included Studies **eFigure 2.** Forest Plots of EV Efficacy (Monotherapy or Combination) in mUC Patients: DCR, ORR, and 1-Year Survival **eFigure 3.** Forest Plots of AEs Incidence for EV (Monotherapy or Combination) in mUC Patients: All-Grade and High-Grade AEs **eFigure 4.** Forest Plots of Specific AEs for EV (Monotherapy or Combination) in mUC Patients: All-Grade and High-Grade AEs **eFigure 5.** Forest Plots of Specific AEs and All-Grade AEs (Overall) for EV (Monotherapy) in mUC Patients **eFigure 6.** Forest Plots of Specific AEs and All-Grade AEs (Overall) for EV+Pembro in mUC Patients **eFigure 7.** Network Diagram of Studies Comparing EV, EV Plus Pembrolizumab, and Chemotherapy in mUC **eTable 2.** CINeMA Assessment of Evidence Certainty for NMA Across Multiple Outcomes (DCR, ORR, and 1-Year Survival) **eFigure 8.** SUCRA Rankings of Treatment Efficacy (A: DCR, B: ORR, C: 1-Year Survival Rate) for mUC: Comparison of EV Plus Pembrolizumab, EV Monotherapy, and Chemotherapy **eFigure 9.** Forest Plots of High-Grade AEs for mUC: Comparison of EV Plus Pembrolizumab, EV Monotherapy, and Chemotherapy **eFigure 10.** SUCRA Rankings of High-Grade AEs for mUC: Comparison of EV Plus Pembrolizumab, EV Monotherapy, and Chemotherapy **eFigure 11.** Forest Plots of All-Grade AEs for mUC: Comparison of EV Plus Pembrolizumab, EV Monotherapy, and Chemotherapy

Supplement 2.
Data Sharing Statement
