# Supplementary material for: Enfortumab Vedotin With or Without Pembrolizumab in Metastatic Urothelial Carcinoma: A Systematic Review and Meta-Analysis
Source: JAMA Netw Open. 2025 Mar 11;8(3):e250250. doi: 10.1001/jamanetworkopen.2025.0250 (PMC11897842; doi:10.1001/jamanetworkopen.2025.0250)
Supplement: Supplement 1. — eAppendix. Search Strategy for Meta-analysis eTable 1. Clinical Parameters and Results of the Trials Incorporated in This Analysis eFigure 1. The RoB2 Assessment (A) and the ROBINS-I Assessment (B) of the Included Studies eFigure 2. Forest Plots of EV Efficacy (Monotherapy or Combination) in mUC Patients: DCR, ORR, and 1-Year Survival eFigure 3. Forest Plots of AEs Incidence for EV (Monotherapy or Combination) in mUC Patients: All-Grade and High-Grade AEs eFigure 4. Forest Plots of Specific AEs for EV (Monotherapy or Combination) in mUC Patients: All-Grade and High-Grade AEs eFigure 5. Forest Plots of Specific AEs and All-Grade AEs (Overall) for EV (Monotherapy) in mUC Patients eFigure 6. Forest Plots of Specific AEs and All-Grade AEs (Overall) for EV+Pembro in mUC Patients eFigure 7. Network Diagram of Studies Comparing EV, EV Plus Pembrolizumab, and Chemotherapy in mUC eTable 2. CINeMA Assessment of Evidence Certainty for NMA Across Multiple Outcomes (DCR, ORR, and 1-Year Survival) eFigure 8. SUCRA Rankings of Treatment Efficacy (A: DCR, B: ORR, C: 1-Year Survival Rate) for mUC: Comparison of EV Plus Pembrolizumab, EV Monotherapy, and Chemotherapy eFigure 9. Forest Plots of High-Grade AEs for mUC: Comparison of EV Plus Pembrolizumab, EV Monotherapy, and Chemotherapy eFigure 10. SUCRA Rankings of High-Grade AEs for mUC: Comparison of EV Plus Pembrolizumab, EV Monotherapy, and Chemotherapy eFigure 11. Forest Plots of All-Grade AEs for mUC: Comparison of EV Plus Pembrolizumab, EV Monotherapy, and Chemotherapy [file jamanetwopen-e250250-s001.pdf]

Supplemental Online Content

Yajima S, Hirose K, Masuda H. Enfortumab vedotin with or without pembrolizumab in metastatic urothelial carcinoma: a meta-analysis. *JAMA Netw Open*. 2025;8(3):e250250. doi:10.1001/jamanetworkopen.2025.0250

eAppendix. Search Strategy for Meta-analysis
eTable 1. Clinical Parameters and Results of the Trials Incorporated in This Analysis
eFigure 1. The RoB2 Assessment (A) and the ROBINS-I Assessment (B) of the Included Studies
eFigure 2. Forest Plots of EV Efficacy (Monotherapy or Combination) in mUC Patients: DCR, ORR, and 1-Year Survival
eFigure 3. Forest Plots of AEs Incidence for EV (Monotherapy or Combination) in mUC Patients: All-Grade and High-Grade AEs
eFigure 4. Forest Plots of Specific AEs for EV (Monotherapy or Combination) in mUC Patients: All-Grade and High-Grade AEs
eFigure 5. Forest Plots of Specific AEs and All-Grade AEs (Overall) for EV (Monotherapy) in mUC Patients
eFigure 6. Forest Plots of Specific AEs and All-Grade AEs (Overall) for EV+Pembro in mUC Patients
eFigure 7. Network Diagram of Studies Comparing EV, EV Plus Pembrolizumab, and Chemotherapy in mUC
eTable 2. CINeMA Assessment of Evidence Certainty for NMA Across Multiple Outcomes (DCR, ORR, and 1-Year Survival)
eFigure 8. SUCRA Rankings of Treatment Efficacy (A: DCR, B: ORR, C: 1-Year Survival Rate) for mUC: Comparison of EV Plus Pembrolizumab, EV Monotherapy, and Chemotherapy
eFigure 9. Forest Plots of High-Grade AEs for mUC: Comparison of EV Plus Pembrolizumab, EV Monotherapy, and Chemotherapy
eFigure 10. SUCRA Rankings of High-Grade AEs for mUC: Comparison of EV Plus Pembrolizumab, EV Monotherapy, and Chemotherapy
eFigure 11. Forest Plots of All-Grade AEs for mUC: Comparison of EV Plus Pembrolizumab, EV Monotherapy, and Chemotherapy

This supplemental material has been provided by the authors to give readers additional information about their work.

© 2025 Yajima S et al. *JAMA Network Open*

eAppendix: Search strategy for meta-analysis

Electronic searches were performed in the following databases to identify eligible studies: There were no language or publication period limitations.

PubMed (n = 105)
((("Carcinoma, Transitional Cell"[Mesh] OR "Urinary Bladder Neoplasms"[Mesh] OR "Urethral Neoplasms"[Mesh] OR "Ureteral Neoplasms"[Mesh] OR "urothelial carcinoma"[tiab] OR "bladder cancer"[tiab]) AND ("Neoplasm Metastasis"[Mesh] OR metasta*[tiab] OR advanced[tiab])) AND ("enfortumab vedotin"[Supplementary Concept] OR "enfortumab vedotin"[tiab] OR "ASG-22CE"[tiab] OR "ASG-22ME"[tiab]) AND (randomized[tiab] OR randomised[tiab] OR "Randomized Controlled Trial"[pt] OR "Clinical Trial"[pt] OR "Controlled Clinical Trial"[pt] OR trial[tiab] OR "Clinical Study"[pt] OR cohort[tiab] OR "Cohort Studies"[Mesh] OR "Observational Study"[pt]))

Cochrane Library (n = 61)
#1 ("urothelial carcinoma" OR "bladder cancer" OR "transitional cell carcinoma" OR "urinary bladder neoplasm*" OR "urethral neoplasm*" OR "ureteral neoplasm*"):ti,ab,kw
#2 (metasta* OR advanced):ti,ab,kw
#3 ("enfortumab vedotin" OR "ASG-22CE" OR "ASG-22ME"):ti,ab,kw
#4 (random* OR trial OR "clinical study" OR cohort OR "observational study"):ti,ab,kw
#5 #1 AND #2 AND #3 AND #4

Web of science (n = 156)
TS=("urothelial carcinoma" OR "bladder cancer" OR "transitional cell carcinoma" OR "urinary bladder neoplasm*" OR "urethral neoplasm*" OR "ureteral neoplasm*")
AND
TS=(metasta* OR advanced)
AND
TS=("enfortumab vedotin" OR "ASG-22CE" OR "ASG-22ME")
AND
TS=(randomized OR randomised OR trial OR "clinical study" OR cohort OR "observational study")

Google scholar (n = 479)
("urothelial carcinoma" OR "bladder cancer" OR "transitional cell carcinoma")
(metastatic OR advanced)
"enfortumab vedotin"
("randomized controlled trial" OR "prospective study" OR "phase II" OR "phase III")
-retrospective -"case report" -review

© 2025 Yajima S et al. *JAMA Network Open*

eTable 1. Clinical parameters and results of the trials incorporated in this analysis

Study name	Age (year), median (range)	Follow-up duration (months)	Line of therapy	Dosage of treatment	Schedule of treatment	Proportion of males, No. (%)	ECOG-PS 0, No. (%)	ECOG-PS 1, No. (%)	Primary tumor site	Metastatic sites	Prior treatment history
EV-302 [11]	69 (37-87)	17.2	First-line	EV 1.25 mg/kg (max 125 mg per dose), pembro 200 mg	3-week cycles, EV on days 1 and 8, pembro on day 1	344 (77.8)	223 (50.5)	204 (46.2)	Lower tract 72.7%, Upper tract 27.0%	Visceral 71.8%, Lymph node only 23.4%, Liver 22.5%, Lung 37.0%, Bone 20.7%	No previous systemic therapy except neoadjuvant or adjuvant chemotherapy with recurrence >12 months after completion
EV-301 [29]	68 (34-85)	23.75	Second-line or later	1.25 mg/kg (maximum weight, 100 kg)	Days 1, 8, and 15 of each 28-day cycle	238 (79.1)	120 (39.9)	181 (60.1)	Upper tract 33.2%, Bladder/other 66.3%	Liver 30.9%, No liver metastasis 69.1%	Prior platinum-containing chemotherapy and disease progression during or after PD-1/L1 inhibitor treatment
EV-103 Cohort K [30]	71 (51-91)	14.8	First-line	EV 1.25 mg/kg (maximum 125 mg), pembro 200 mg	EV on days 1 and 8, pembro on day 1 of 3-week cycles	54 (71.1)	33 (43.4)	33 (43.4)	Lower tract 60.5%, Upper tract 39.5%	Visceral 84.2%, Liver 17.1%, Lung 48.7%, Bone	No previous systemic treatment for locally advanced or metastatic disease

© 2025 Yajima S et al. *JAMA Network Open*

										25.0%	
EV-101 [31]	67 (24-86)	16.4	Second-line or later	1.25 mg/kg	Days 1, 8, and 15 of 28-day cycles	111 (71.6)	46 (29.7)	109 (70.3)[a]	Bladder 71%, Upper tract 25%	Visceral 77%, Lung 51%, Liver 39%	96% prior platinum-based chemotherapy, 72% prior anti-PD-(L)1 treatment, 35% prior taxane therapy
EV-201 Cohort 1 [32]	69 (40-84)	10.2	Second-line or later	1.25 mg/kg	Days 1, 8, and 15 of 28-day cycles	88 (70.4)	40 (32.0)	85 (68.0)	Bladder/other 64.8%, Upper tract 35.2%	Visceral 89.6%, Liver 40%, Lung 42.4%, Bone 40.8%	Median 3 prior systemic therapies (range 1-6), 100% prior platinum-based chemotherapy, 100% prior PD-1/L1 inhibitor
EV-201 Cohort 2 [33]	75 (49-90)	13.4	Second-line	1.25 mg/kg	Days 1, 8, and 15 of 28-day cycles	66 (74.2)	37 (41.6)	41 (46.1)	Bladder/other 57.30%, Upper tract 42.70%	Visceral 78.65%, Lymph nodes only 20.22%, Liver 23.60%, Lung 46.07%, Bone 24.72%	100% prior PD-1/PD-L1 inhibitor
DAD [34]	70 (41-88)	14	Second-line or later	Sacituzumab govitecan 8 mg/kg and EV 1.25 mg/kg (recommended phase II dose)	Days 1 and 8 of 21-day cycle	18 (78.3)	14 (60.9)	9 (39.1)	Bladder 69.57%, Upper tract 26.09%, Urethra 4.35%	Lymph nodes 73.91%, Liver 26.09%, Bone 26.09%, Lung 21.74%, Kidney 13.04%	96% prior immunotherapy, 78% prior cisplatin-based chemotherapy, 26% prior carboplatin-based chemotherapy
NA	71	8.6	Third-	1.25 mg/kg	Days 1, 8, and 15	25 (73.5)	29	NA	Bladder 47.06%,	NA	100% prior platinum-

© 2025 Yajima S et al. *JAMA Network Open*

(Permission number: 2021-214) [35]	(NA)		line		of 28-day cycle		(85.3)[b]		Upper urinary tract 52.94%		containing chemotherapy and PD-1/PD-L1 inhibitor
EV-102 [36]	67 (57-82)	NA	Second-line or later	EV 1.0 mg/kg (Arm A, n=9) and 1.25 mg/kg (Arm B, n=8)	Days 1, 8, and 15 of each 28-day cycle	15 (88.2)	13 (76.5)	4 (23.5)	Bladder (70.6%), Renal pelvis (17.6%), Ureter (11.8%)	Bone (35.3%), Liver (11.8%), Lung (35.3%), Adrenal gland (11.8%), Brain (5.9%)	All patients had prior cisplatin-based treatment; one patient previously treated with immune checkpoint inhibitor
EV-103 Cohort A [37]	69 (51-90)	47	First-line	EV 1.25 mg/kg	EV on Days 1, 8 and pembrolizumab on Day 1 of 3-week cycles	NA	NA	NA	NA	NA	No previous systemic treatment, cisplatin-ineligible patients
EV-203 [38]	NA	6.5	Second-line or later	1.25 mg/kg	Days 1, 8, and 15 of each 28-day cycle	NA	NA	NA	NA	NA	Previously treated with platinum-based chemotherapy and PD-1/L1 inhibitor therapy

ECOG-PS = Eastern Cooperative Oncology Group Performance Status, EV = Enfortumab Vedotin, Pembro = Pembrolizumab

Annotation: The numerical values in the RCT study are reported exclusively for the treatment group. a: The data is consolidated and presented as PS1 or higher, b: The data is consolidated and presented as PS1 or lower

© 2025 Yajima S et al. *JAMA Network Open*

eFigure 1. The RoB2 assessment (A) and the ROBINS-I assessment (B) of the included studies

(A) RoB2 assessment

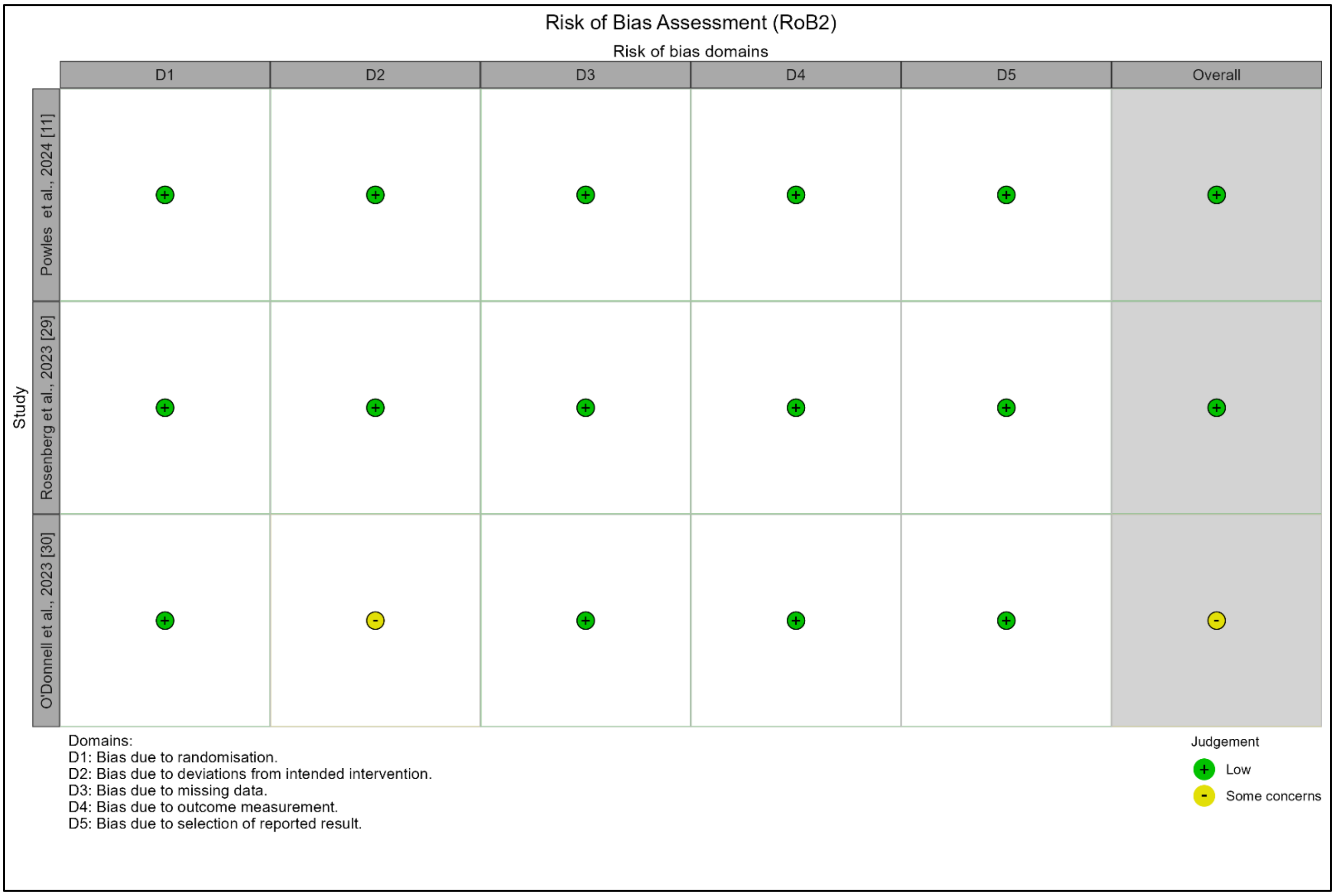

© 2025 Yajima S et al. *JAMA Network Open*

(B) ROBINS-I assessment

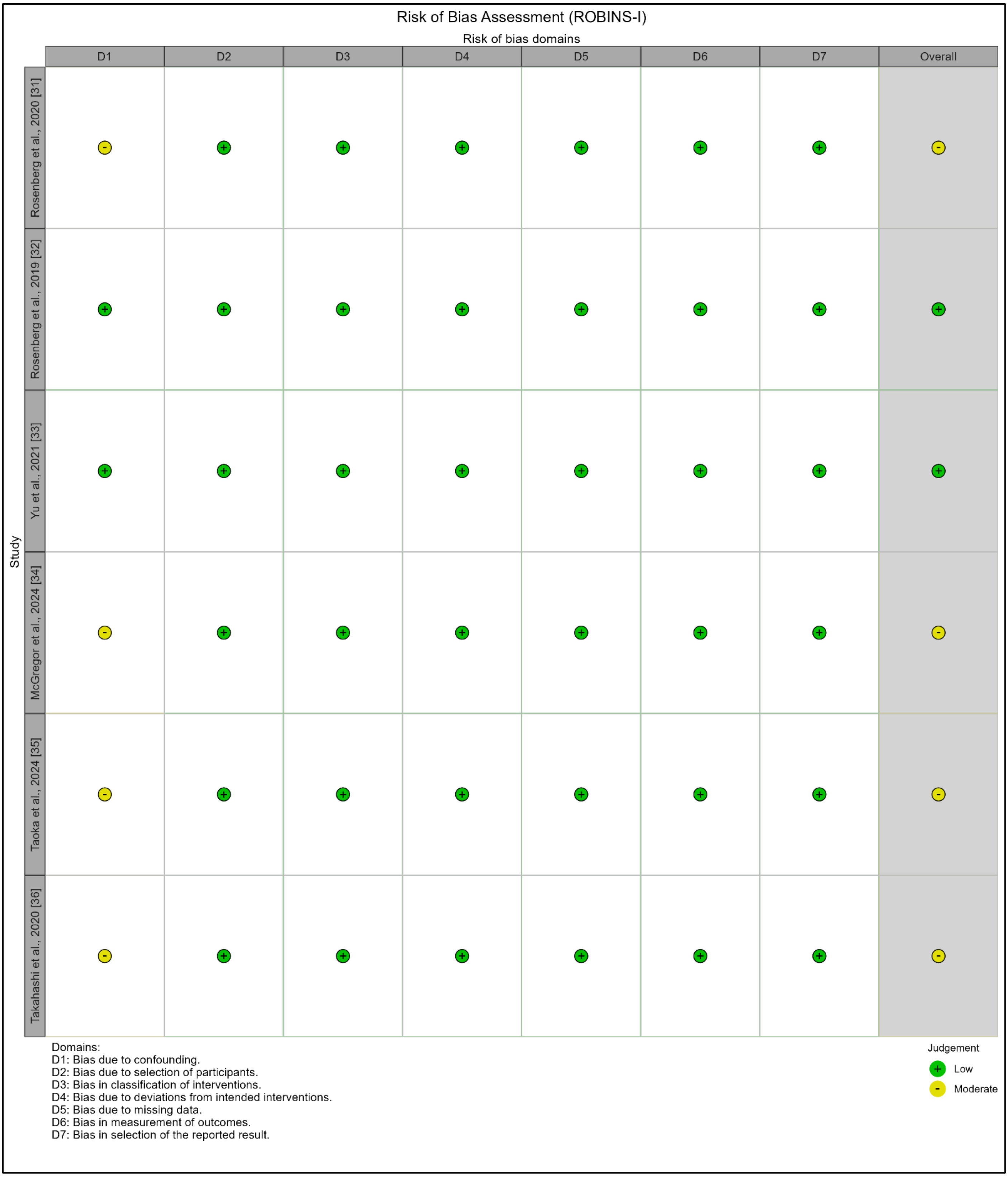

© 2025 Yajima S et al. *JAMA Network Open*

eFigure 2. Forest plots of EV efficacy (Monotherapy or Combination) in mUC patients: DCR (A), ORR (B), and 1-Year Survival (C)

(A) DCR

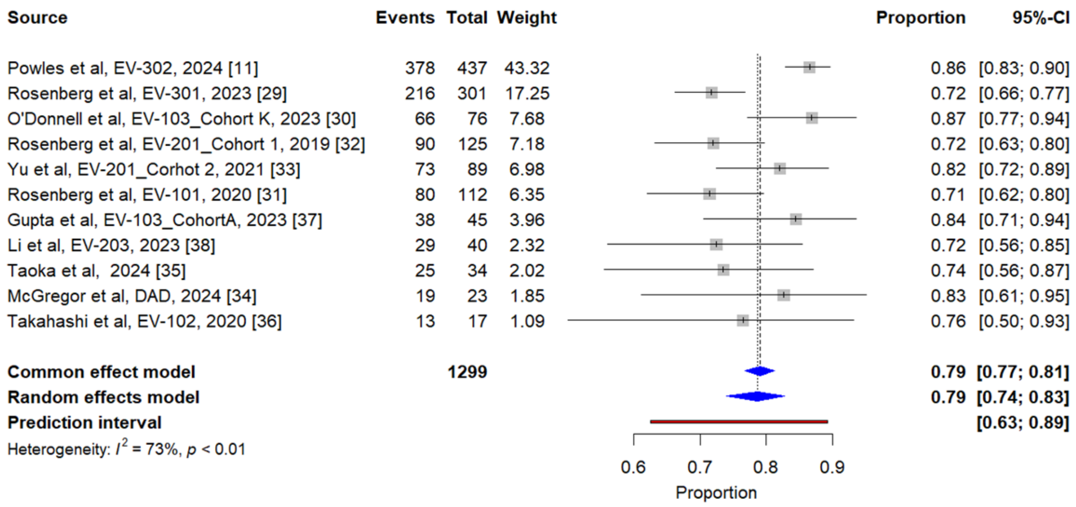

(B) ORR

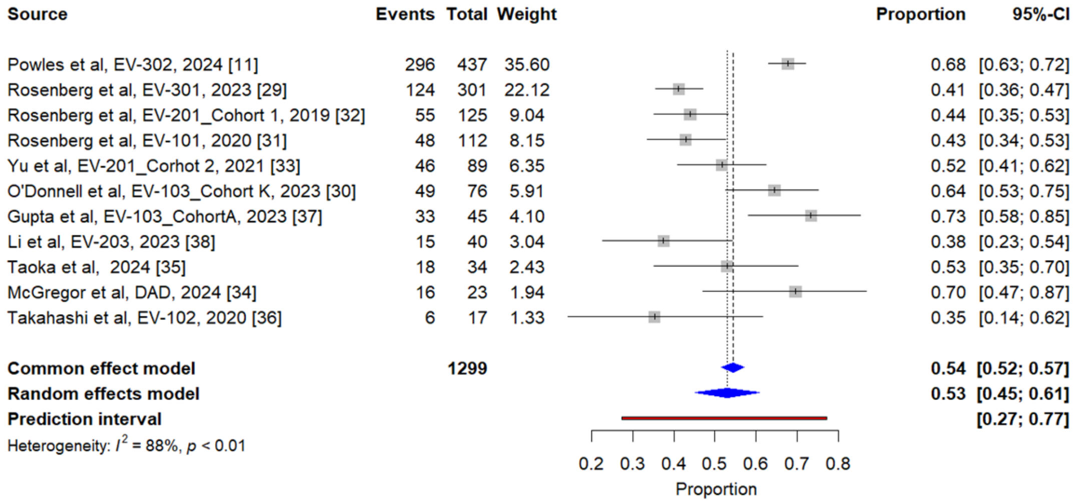

(C) 1-year survival

© 2025 Yajima S et al. *JAMA Network Open*

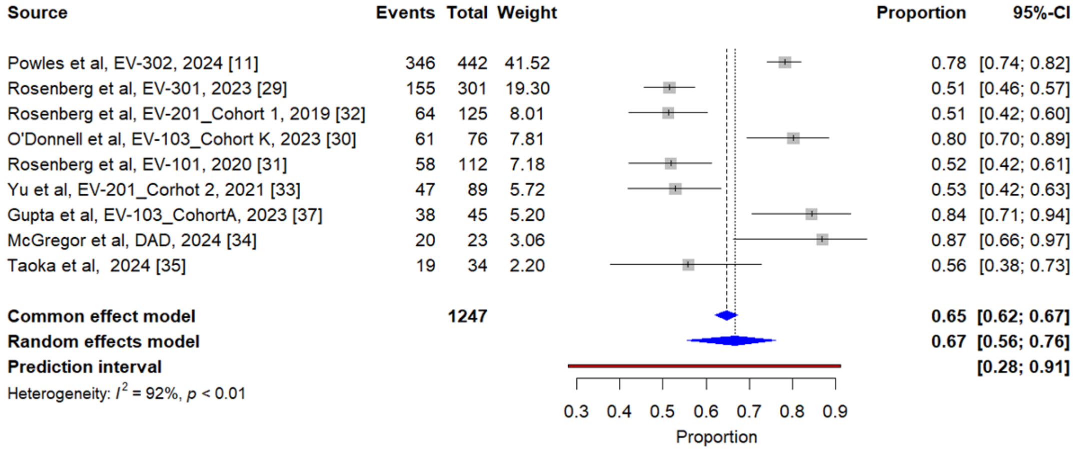

© 2025 Yajima S et al. *JAMA Network Open*

eFigure 3. Forest plots of AEs incidence for EV (Monotherapy or Combination) in mUC patients: All-Grade (A) and High-Grade (B) AEs

(A) All-Grade

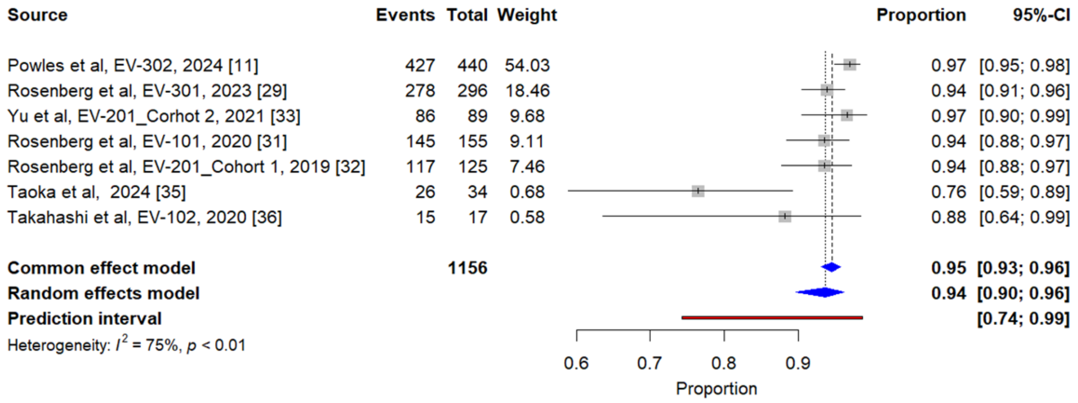

(B) High-Grade

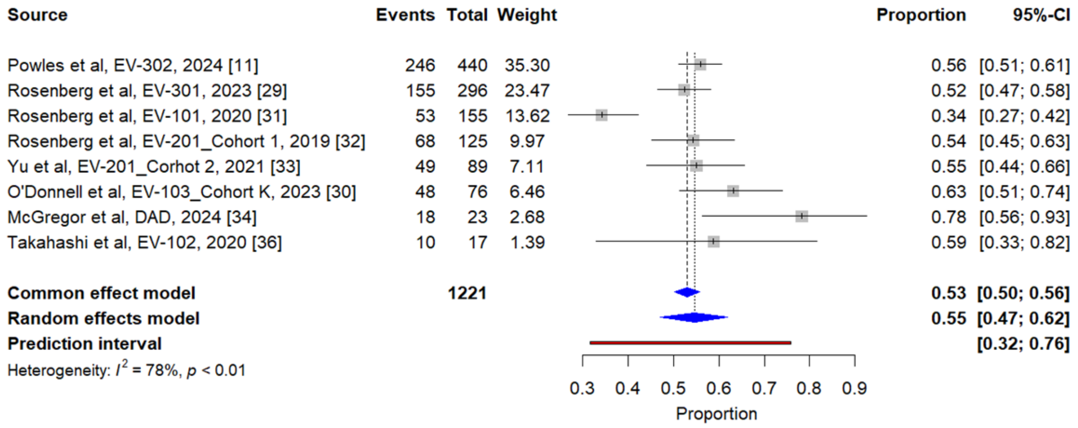

© 2025 Yajima S et al. *JAMA Network Open*

eFigure 4. Forest plots of specific AEs for EV (Monotherapy or Combination) in mUC patients: All-Grade and High-Grade AEs

(A) Anemia (All-Grade)

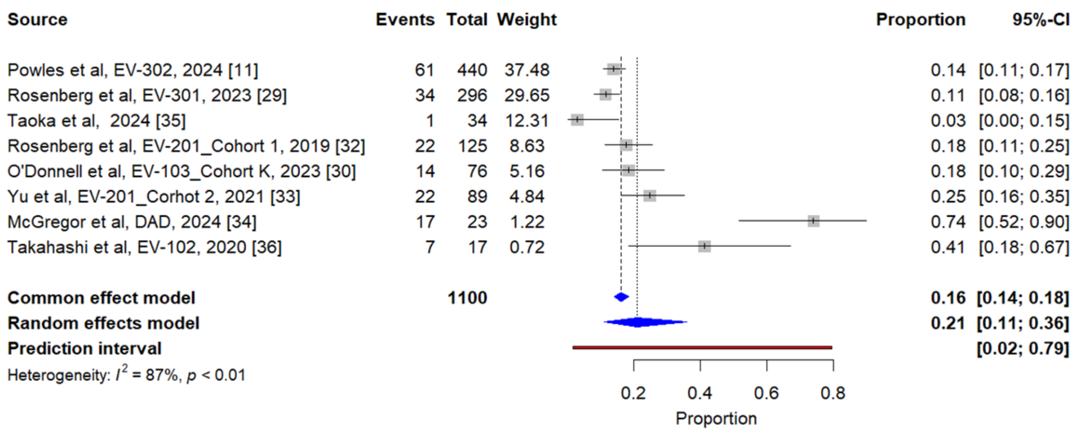

(B) Anemia (High-Grade)

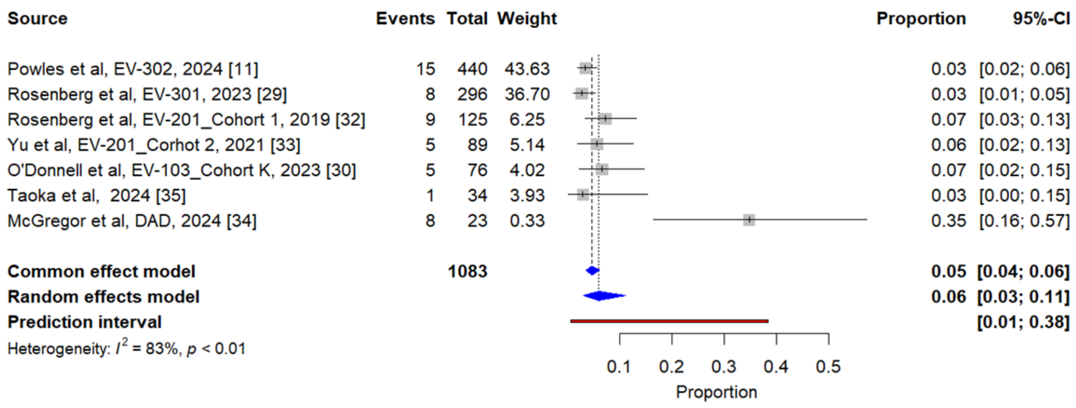

(C) Diarrhea (All-Grade)

© 2025 Yajima S et al. *JAMA Network Open*

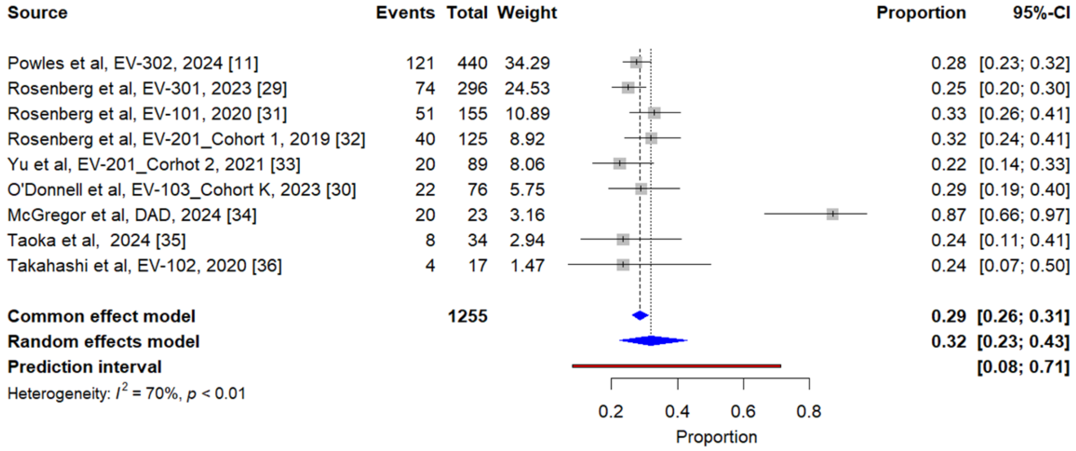

(D) Diarrhea (High-Grade)

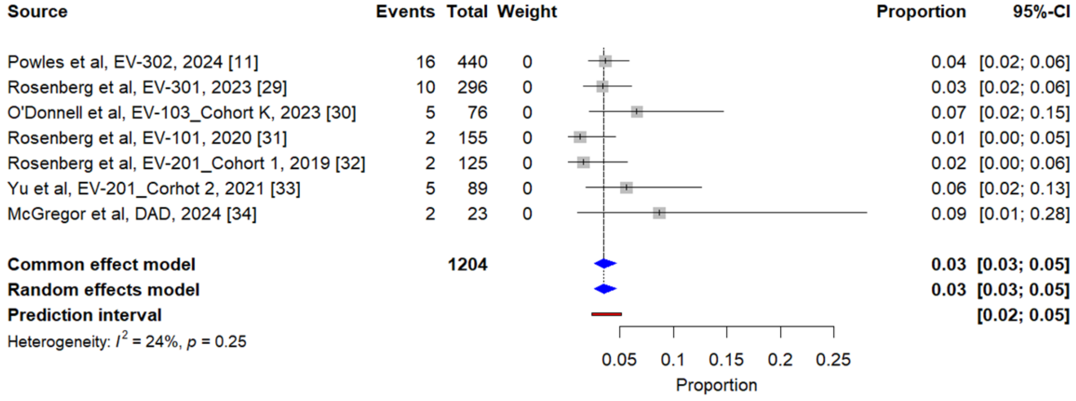

(E) Fatigue (All-Grade)

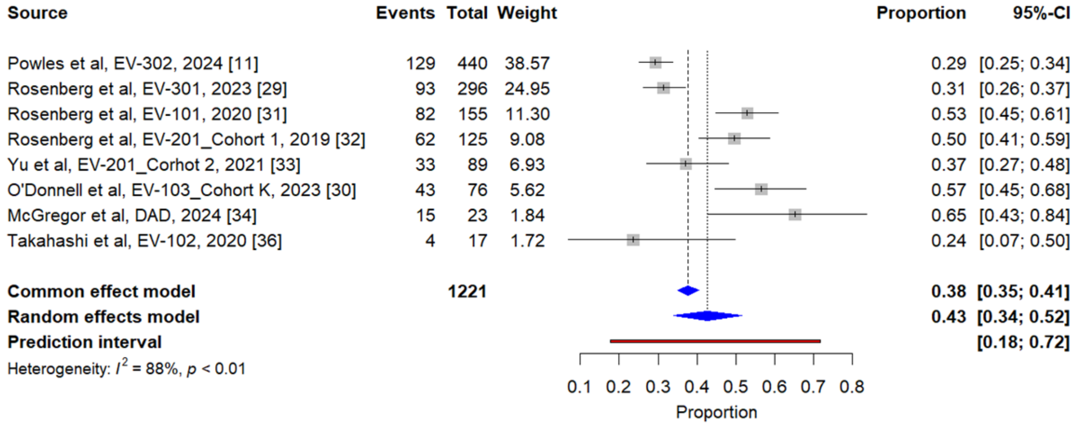

© 2025 Yajima S et al. *JAMA Network Open*

(F) Fatigue (High-Grade)

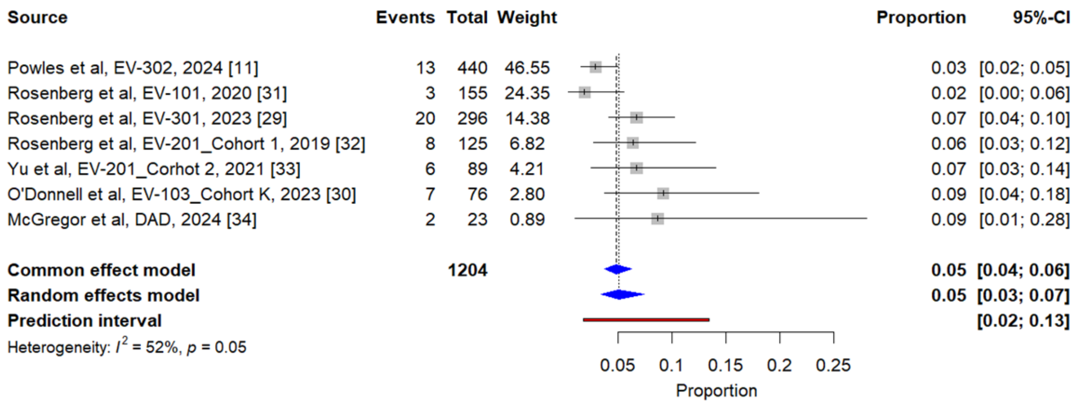

(G) Hyperglycemia (All-Grade)

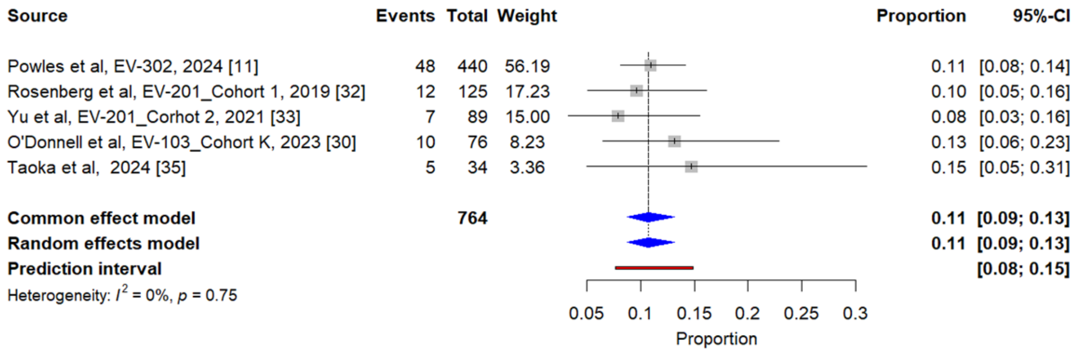

(H) Hyperglycemia (High-Grade)

© 2025 Yajima S et al. *JAMA Network Open*

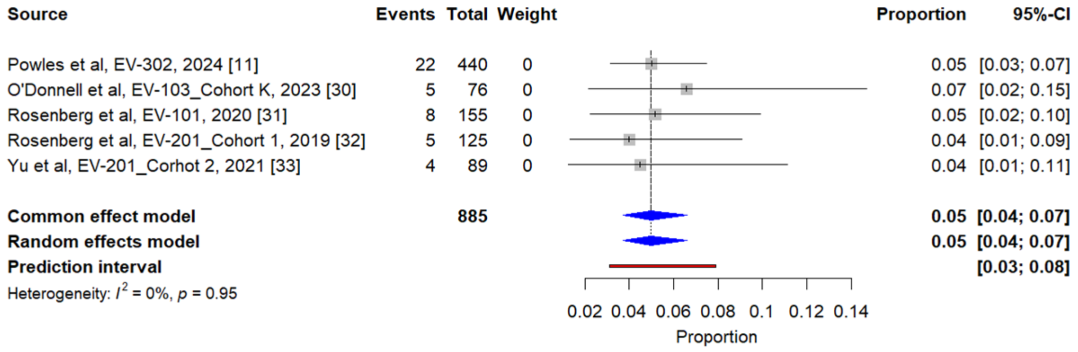

(I) Maculopapular rash (All-Grade)

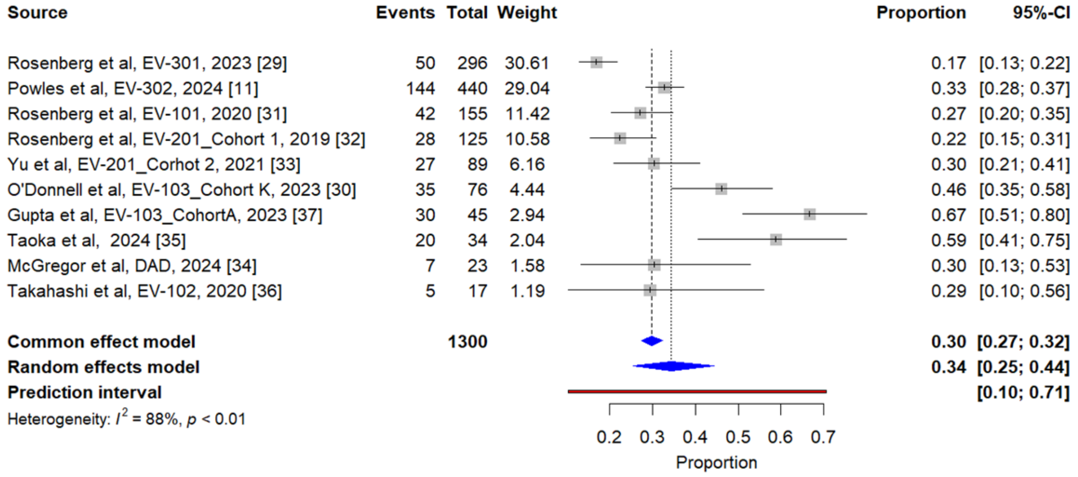

(J) Maculopapular rash (High-Grade)

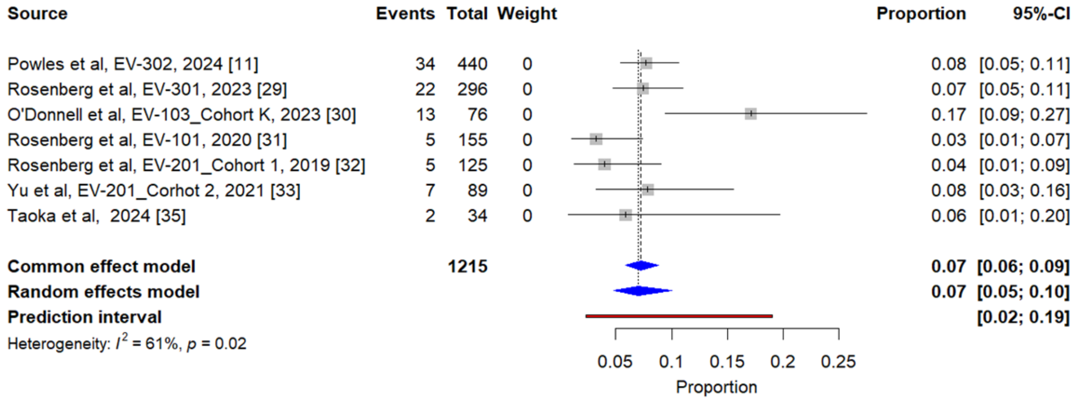

© 2025 Yajima S et al. *JAMA Network Open*

(K) Neutropenia (All-Grade)

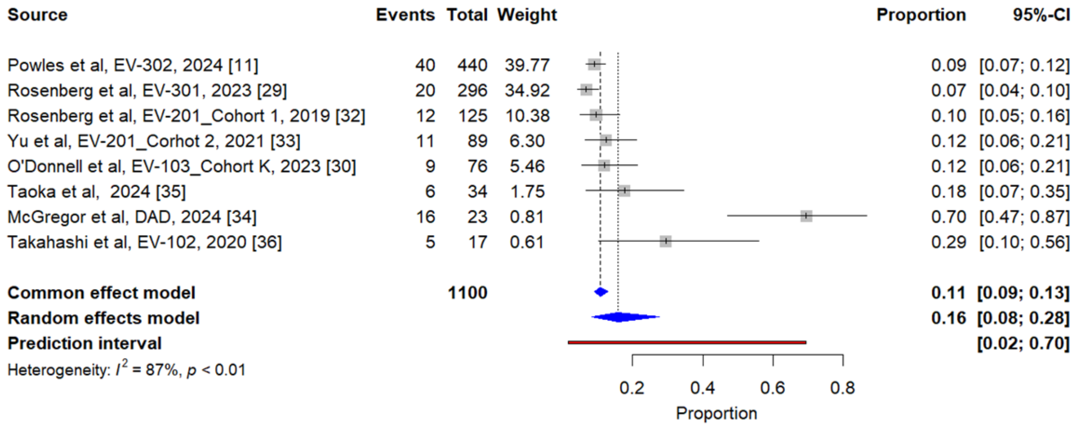

(L) Neutropenia (High-Grade)

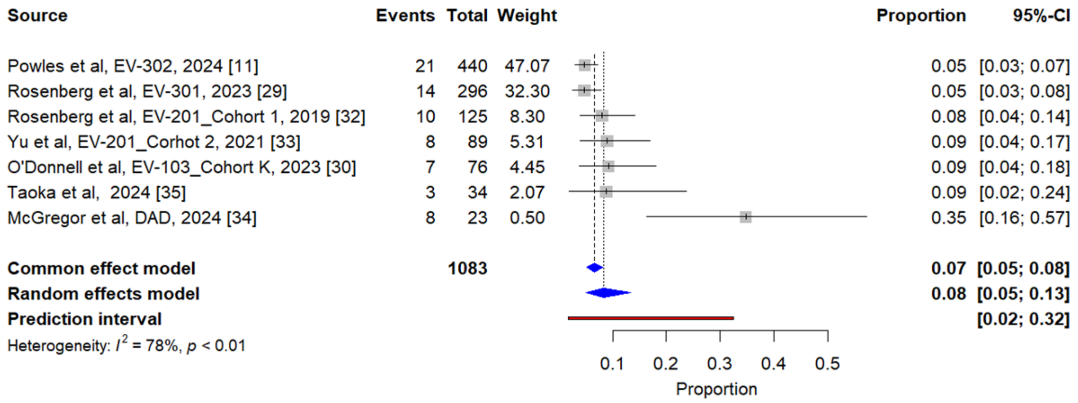

© 2025 Yajima S et al. *JAMA Network Open*

(M) Peripheral neuropathy (All-Grade)

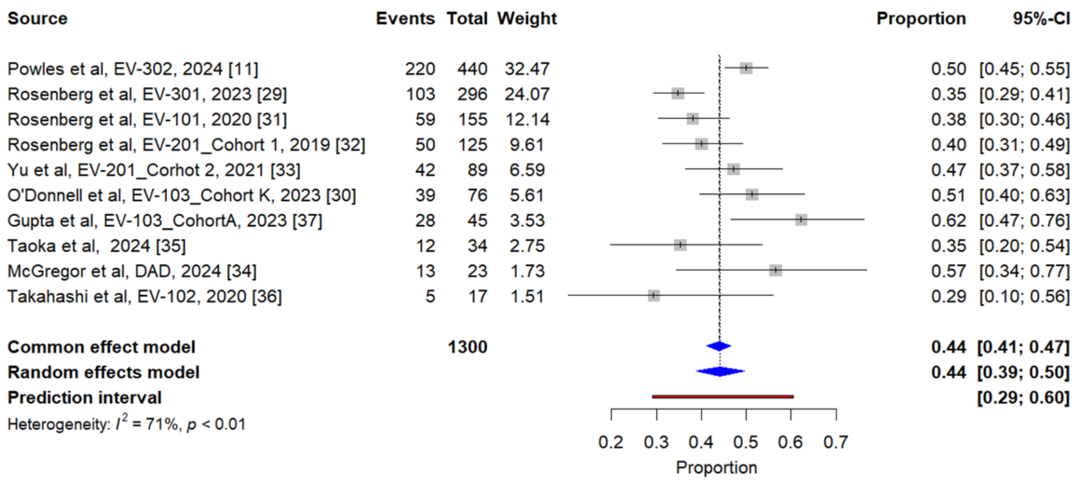

(N) Peripheral neuropathy (High-Grade)

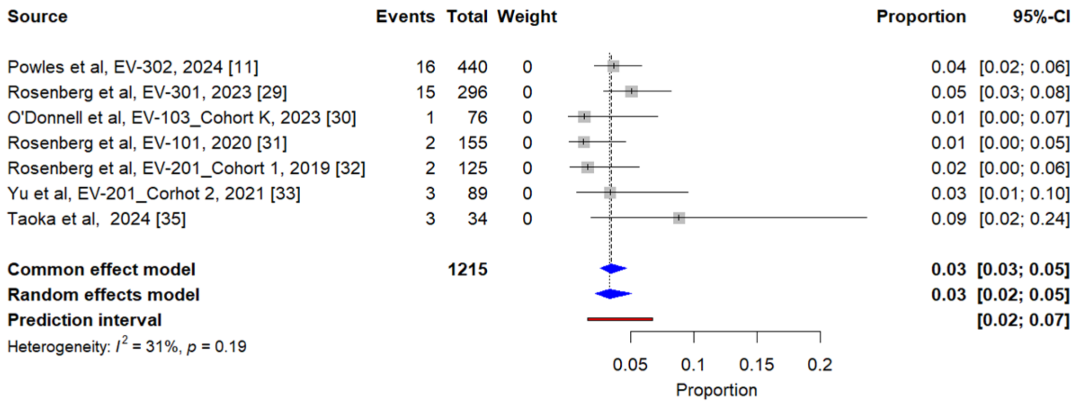

© 2025 Yajima S et al. *JAMA Network Open*

eFigure 5. Forest plots of specific AEs and all-grade AEs (overall) for EV (Monotherapy) in mUC patients

(A) Peripheral neuropathy (All-Grade)

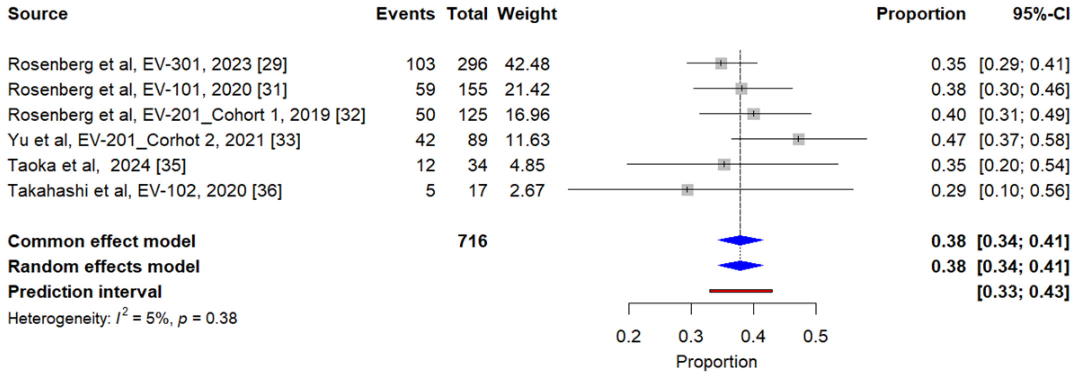

(B) Peripheral neuropathy (High-Grade)

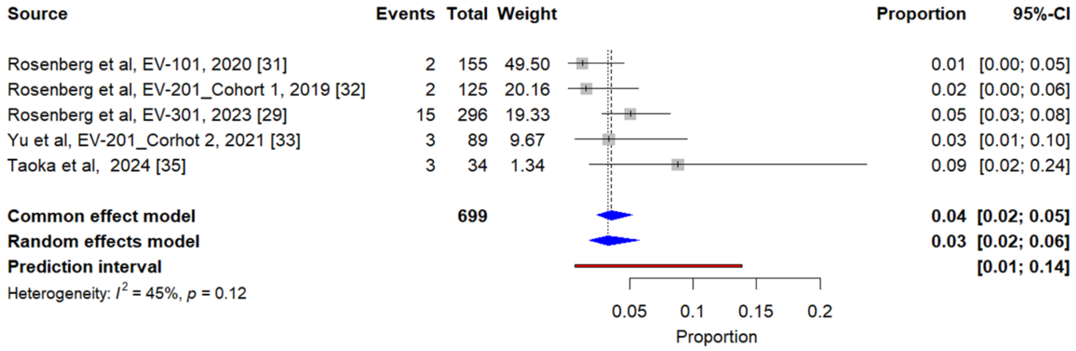

© 2025 Yajima S et al. *JAMA Network Open*

(C) Pruritus (All-Grade)

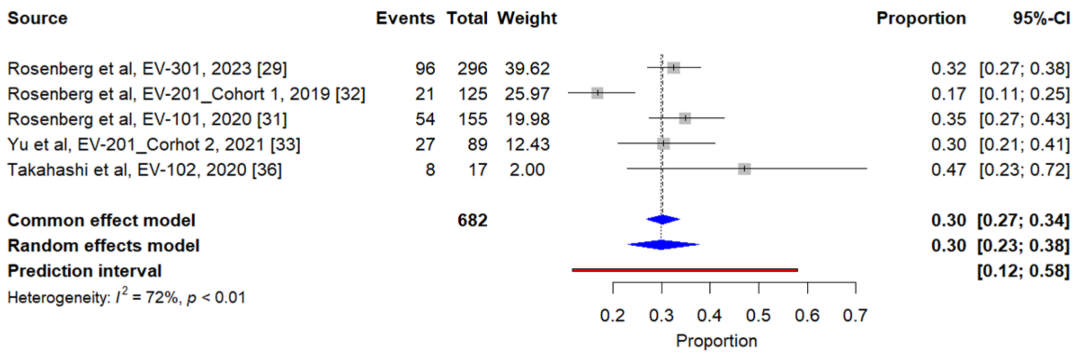

(D) Pruritus (High-Grade)

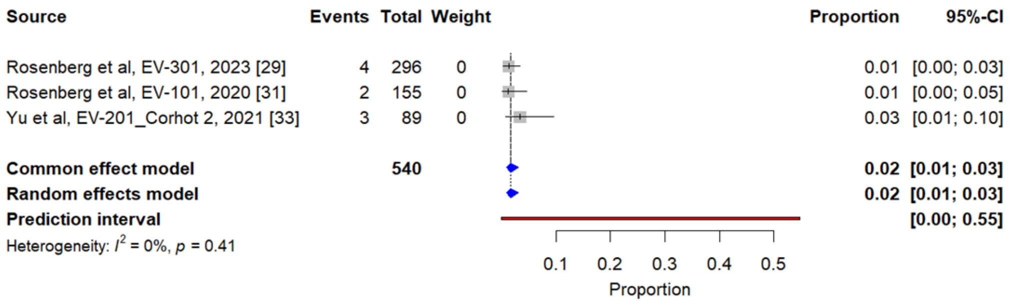

(E) All-grade AEs (overall)

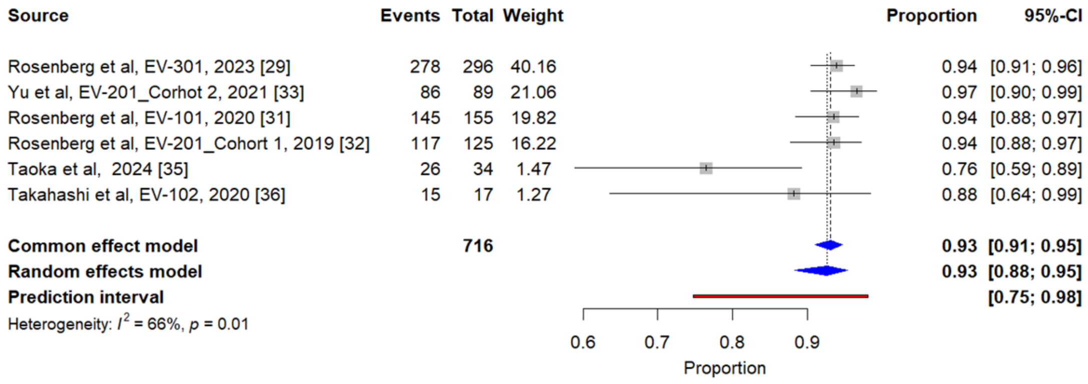

© 2025 Yajima S et al. *JAMA Network Open*

eFigure 6. Forest plots of specific AEs and all-grade AEs (overall) for EV+Pembro in mUC patients

(A) Anemia (All-Grade)

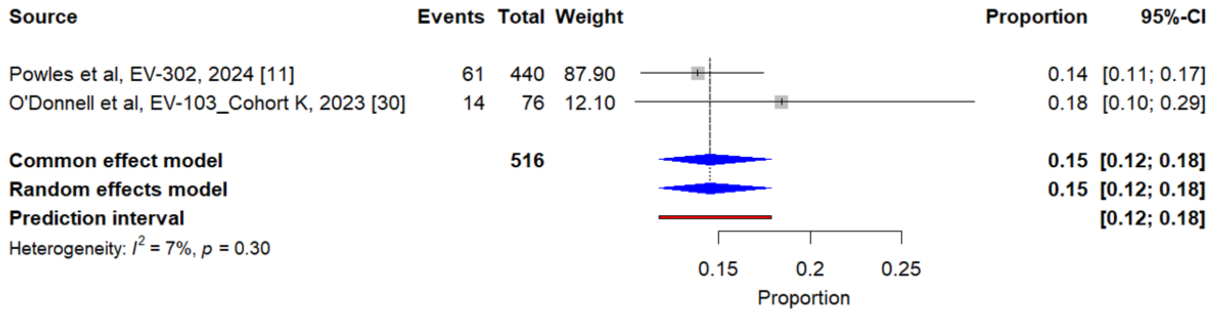

(B) Anemia (High-Grade)

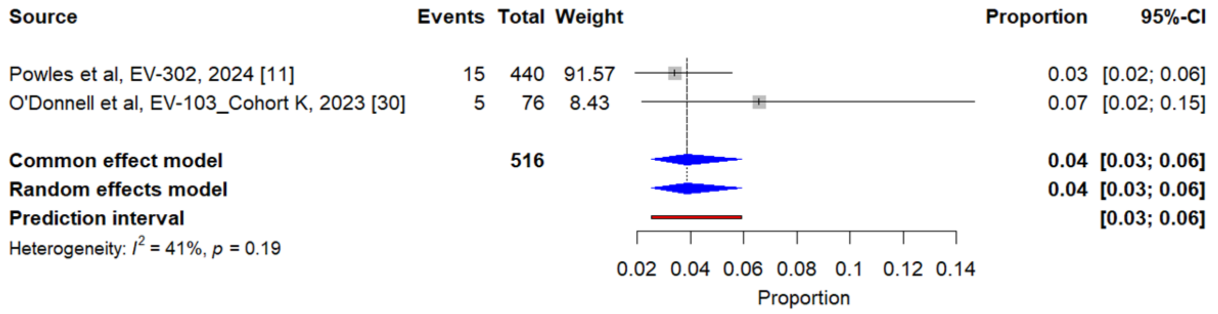

(C) Diarrhea (All-Grade)

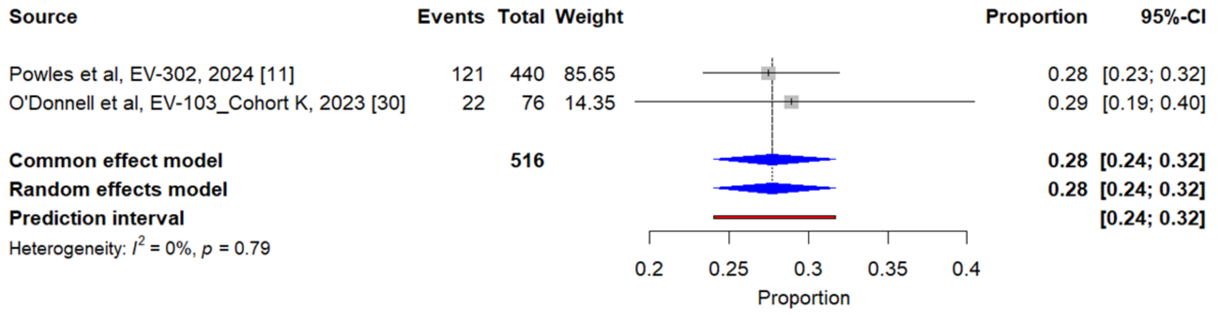

© 2025 Yajima S et al. *JAMA Network Open*

(D) Diarrhea (High-Grade)

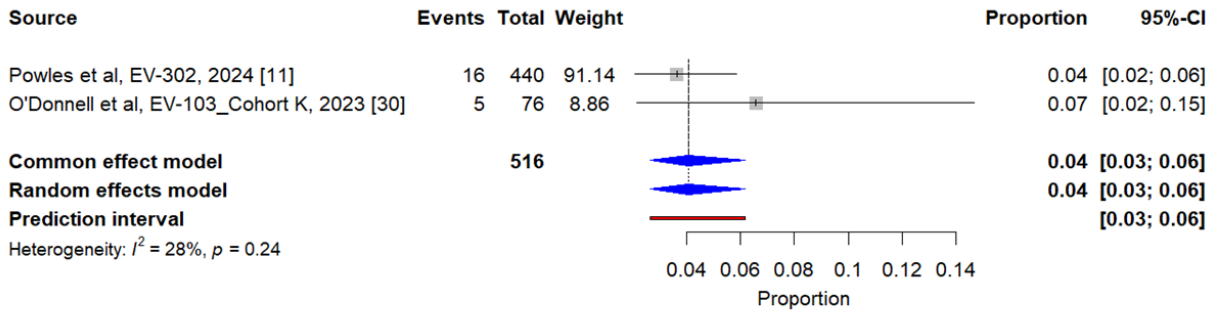

(E) Fatigue (All-Grade)

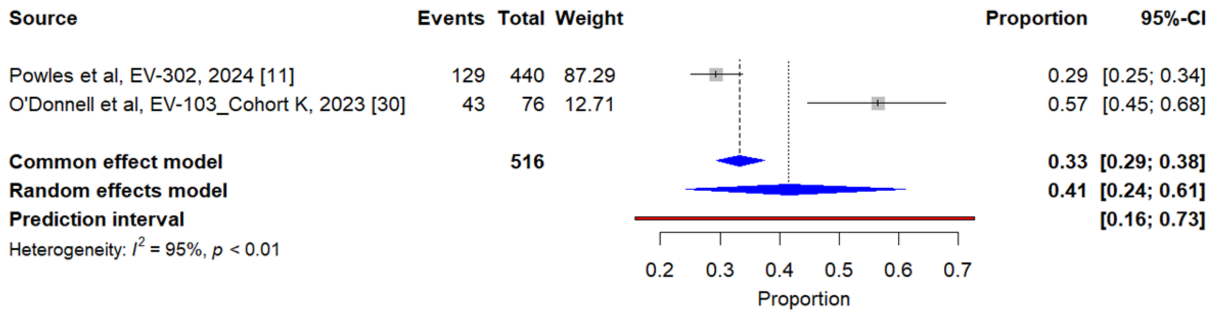

(F) Fatigue (High-Grade)

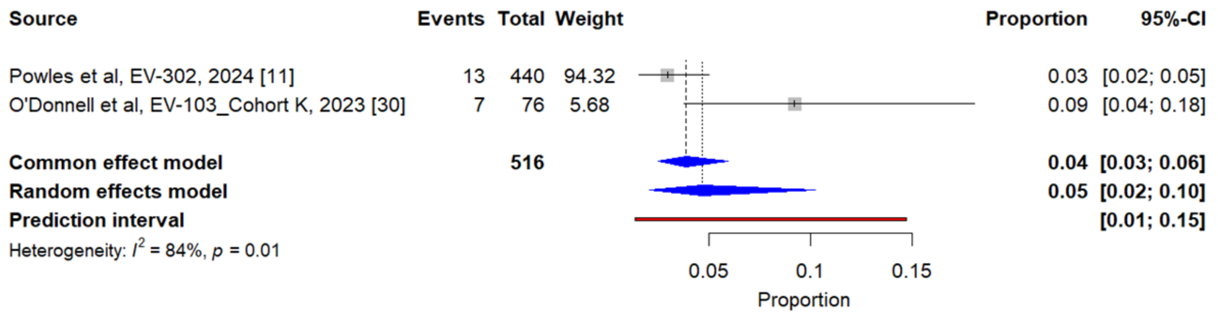

© 2025 Yajima S et al. *JAMA Network Open*

(G) Hyperglycemia (All-Grade)

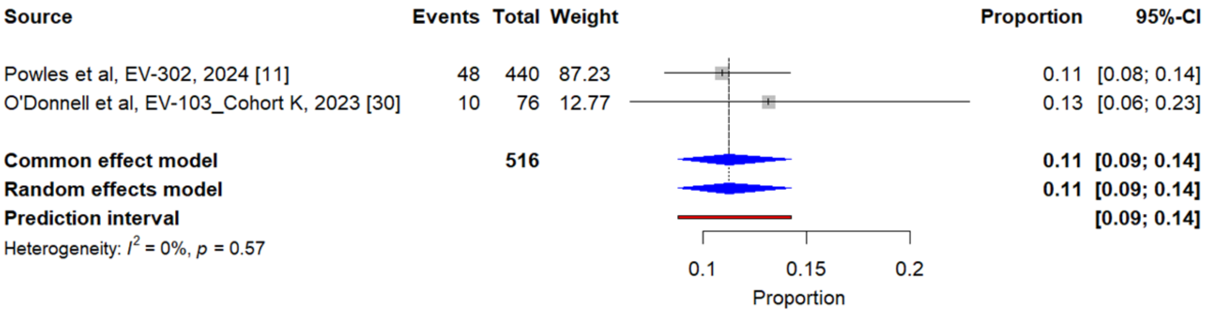

(H) Hyperglycemia (High-Grade)

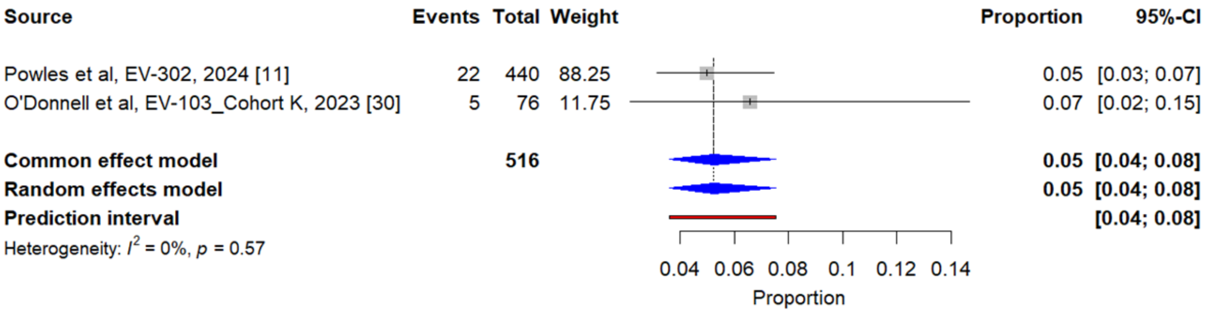

(I) Maculopapular rash (All-Grade)

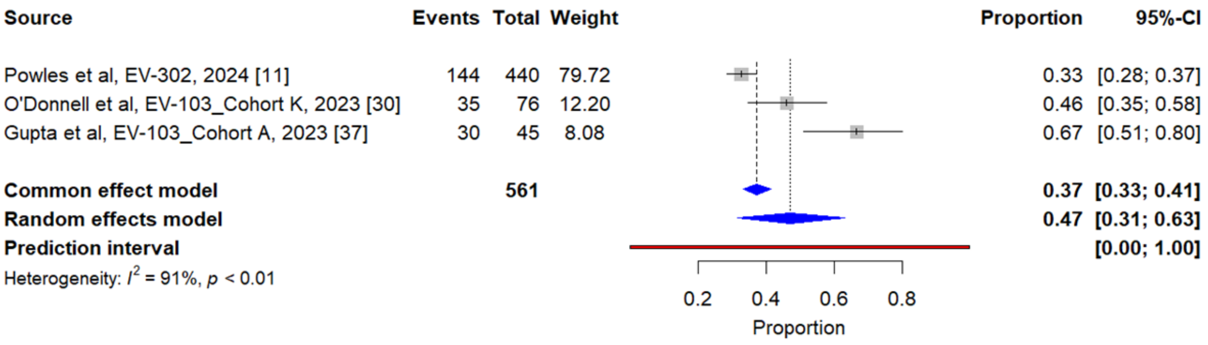

© 2025 Yajima S et al. *JAMA Network Open*

(J) Maculopapular rash (High-Grade)

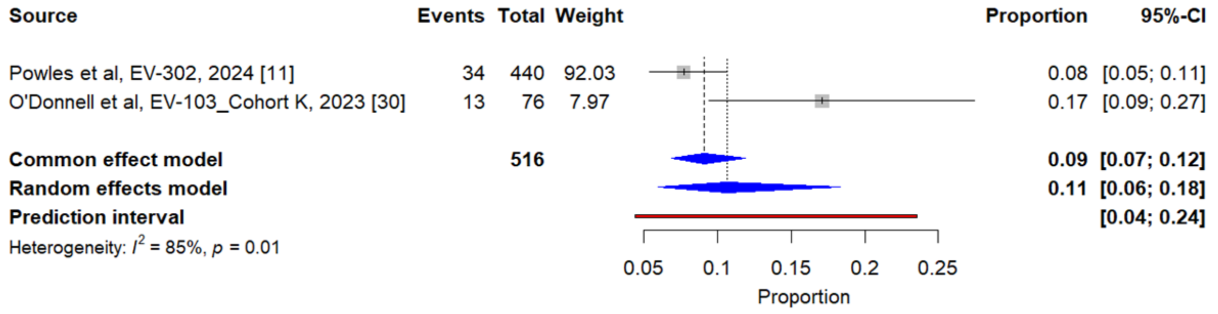

(K) Neutropenia (All-Grade)

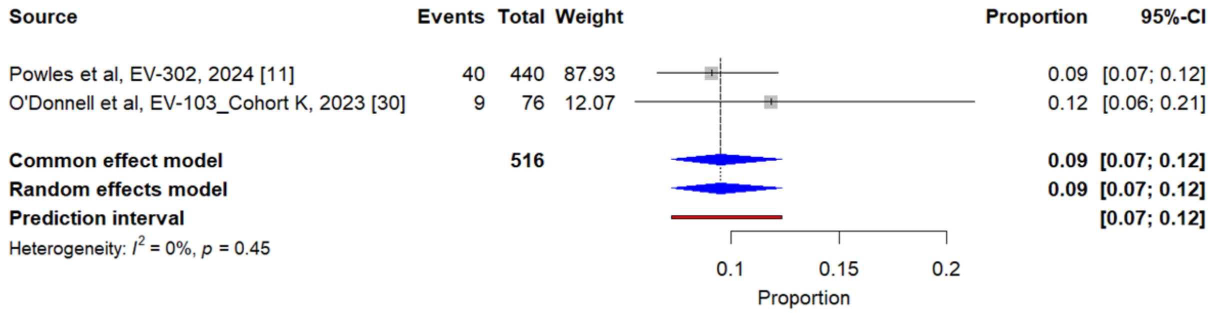

(L) Neutropenia (High-Grade)

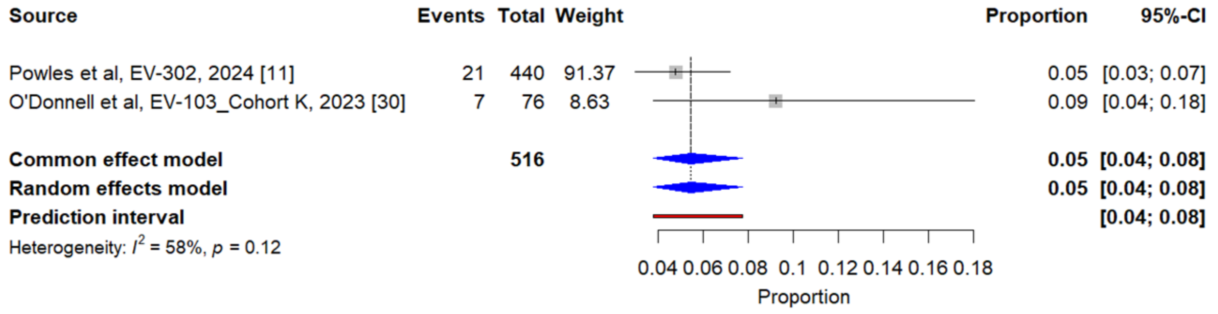

© 2025 Yajima S et al. *JAMA Network Open*

(M) Peripheral neuropathy (All-Grade)

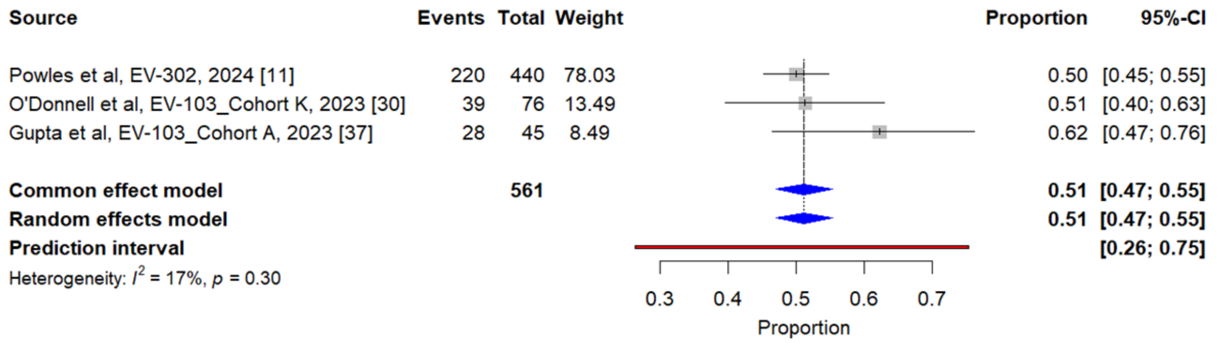

(N) Peripheral neuropathy (High-Grade)

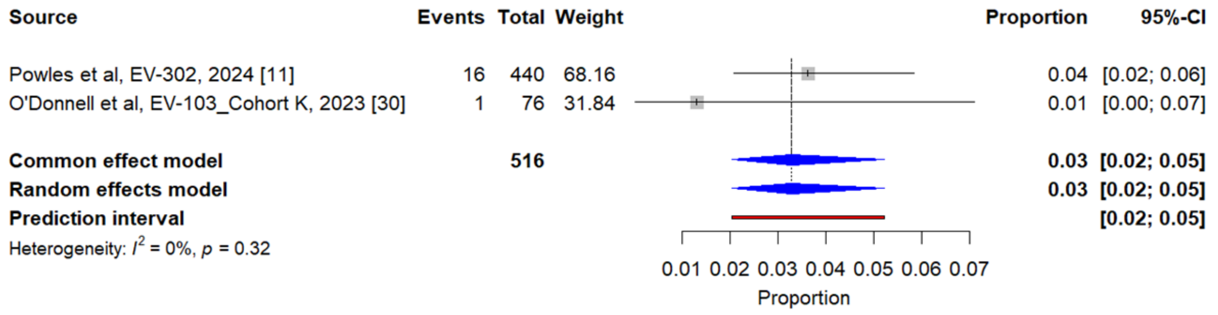

(O) Pruritus (All-Grade)

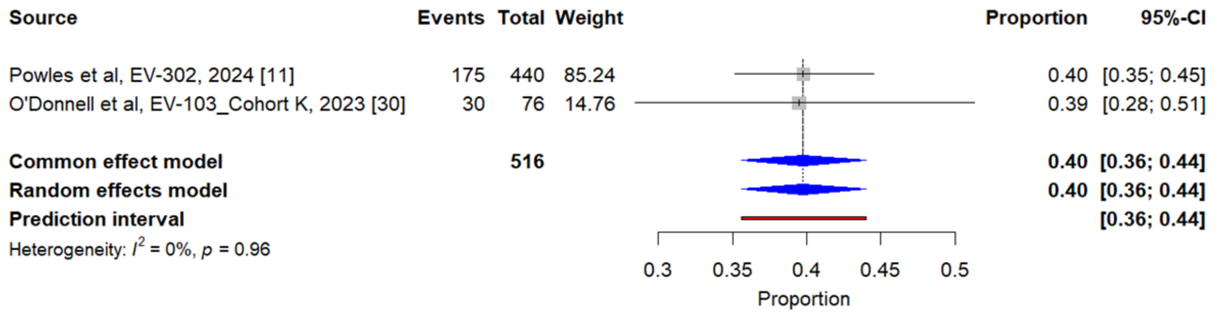

© 2025 Yajima S et al. *JAMA Network Open*

(P) Pruritus (High-Grade)

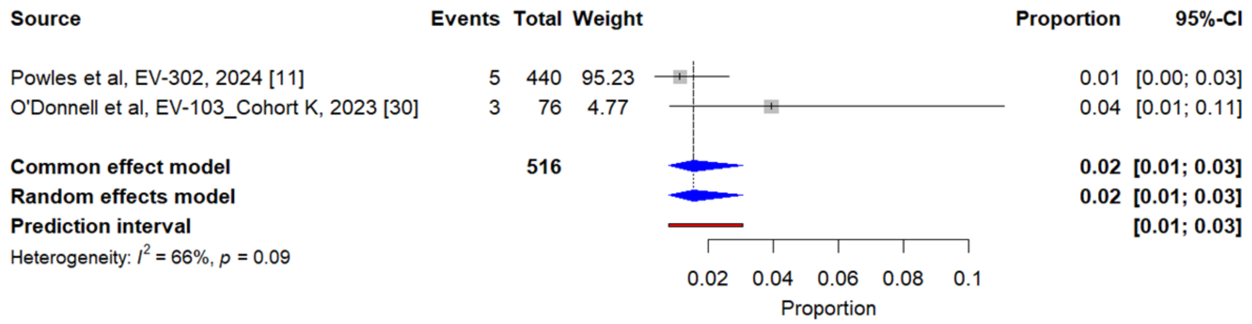

(Q) All-grade AEs (overall)

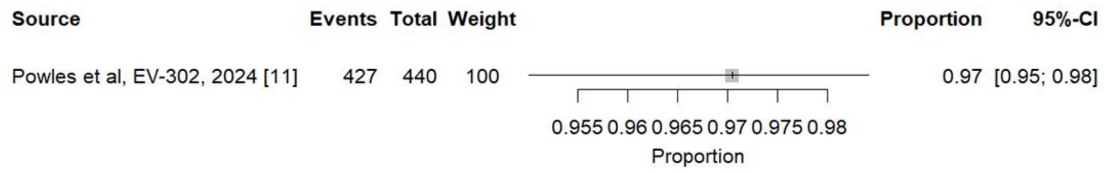

© 2025 Yajima S et al. *JAMA Network Open*

eFigure 7. Network diagram of studies Comparing EV, EV plus Pembrolizumab, and Chemotherapy in mUC

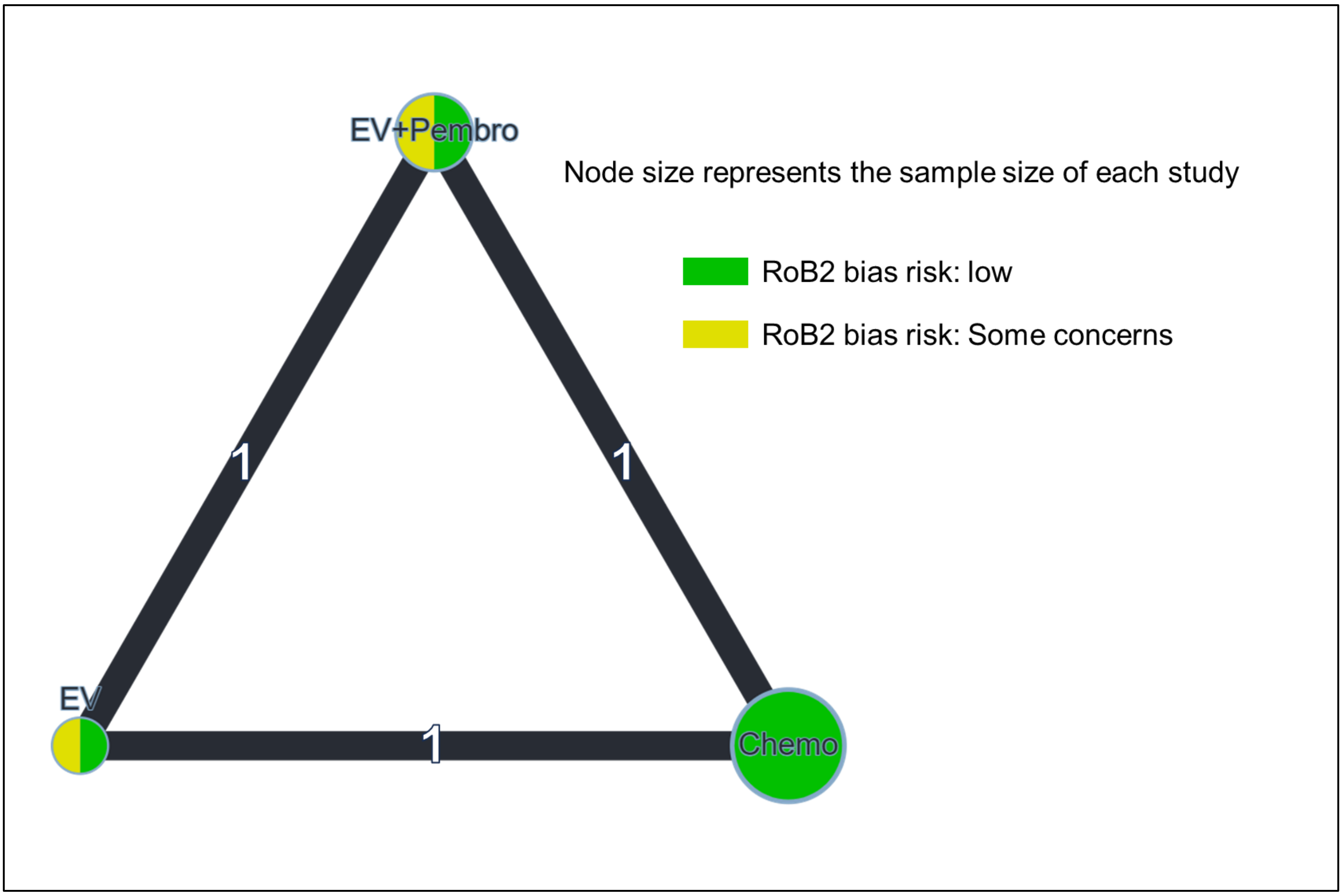

© 2025 Yajima S et al. *JAMA Network Open*

eTable 2. CINeMA assessment of evidence certainty for NMA across multiple outcomes (DCR, ORR, and 1-year survival)

(A) DCR

Comparison	No. of studies	Within-study bias	Reporting bias	Indirectness	Imprecision	Heterogeneity	Incoherence	Confidence rating
Chemo:EV	1	No concerns	No concerns	No concerns	No concerns	Major concerns	No concerns	Low
Chemo:EV+Pembro	1	No concerns	No concerns	No concerns	No concerns	Major concerns	No concerns	Low
EV:EV+Pembro	1	No concerns	No concerns	No concerns	Some concerns	Some concerns	No concerns	Moderate

(B) ORR

Comparison	No. of studies	Within-study bias	Reporting bias	Indirectness	Imprecision	Heterogeneity	Incoherence	Confidence rating
Chemo:EV	1	No concerns	No concerns	No concerns	Some concerns	Some concerns	No concerns	Moderate
Chemo:EV+Pembro	1	No concerns	No concerns	No concerns	No concerns	Major concerns	No concerns	Low
EV:EV+Pembro	1	No concerns	No concerns	No concerns	Some concerns	Some concerns	No concerns	Moderate

(C) 1-year survival

Comparison	No. of studies	Within-study bias	Reporting bias	Indirectness	Imprecision	Heterogeneity	Incoherence	Confidence rating
Chemo:EV	1	No concerns	No concerns	No concerns	No concerns	Major concerns	No concerns	Low
Chemo:EV+Pembro	1	No concerns	No concerns	No concerns	No concerns	Major concerns	No concerns	Low
EV:EV+Pembro	1	No concerns	No concerns	No concerns	Some concerns	Some concerns	No concerns	Moderate

© 2025 Yajima S et al. *JAMA Network Open*

eFigure 8. SUCRA rankings of treatment efficacy (A: DCR, B: ORR, C: 1-year survival rate) for mUC: comparison of EV plus Pembrolizumab, EV monotherapy, and Chemotherapy

(A) DCR

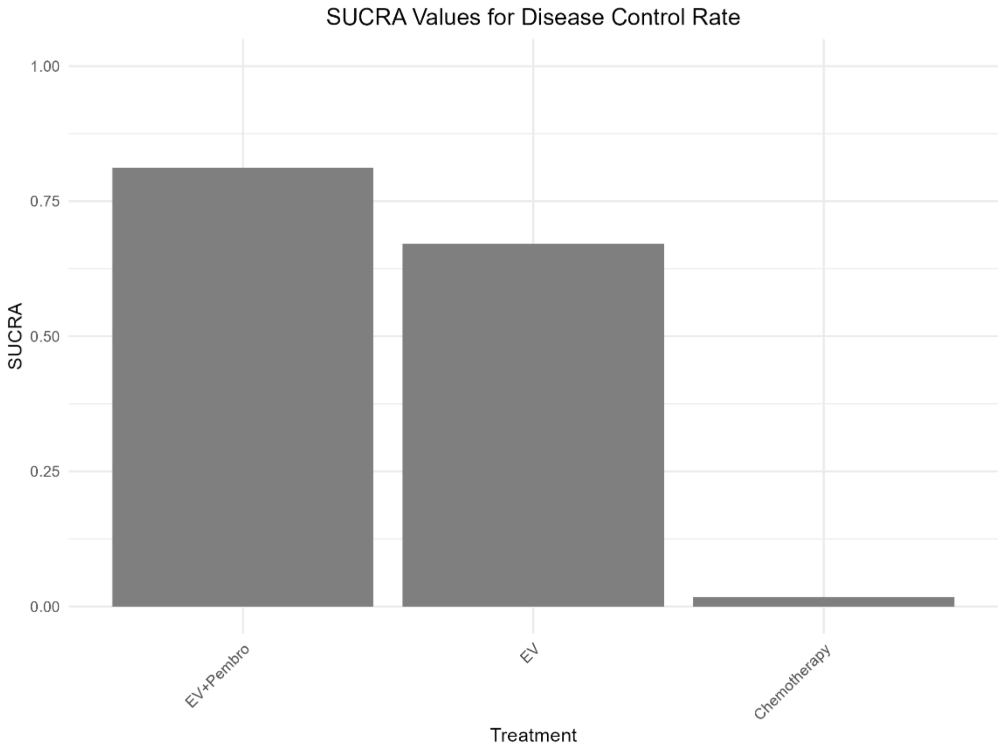

(B) ORR

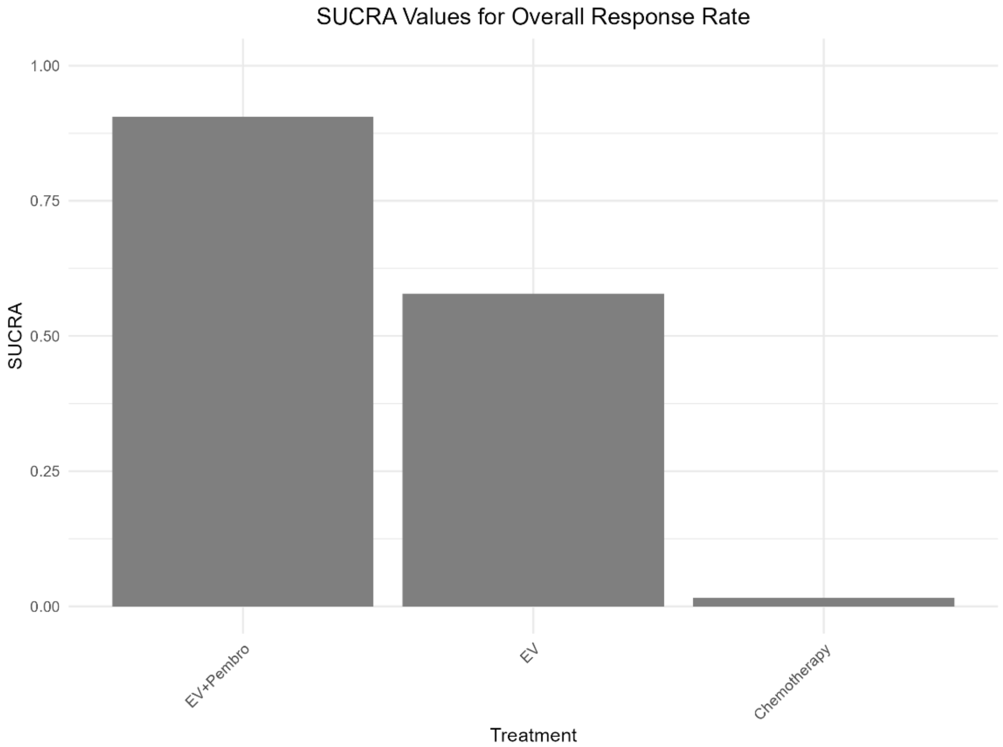

© 2025 Yajima S et al. *JAMA Network Open*

(C) 1-year survival rate

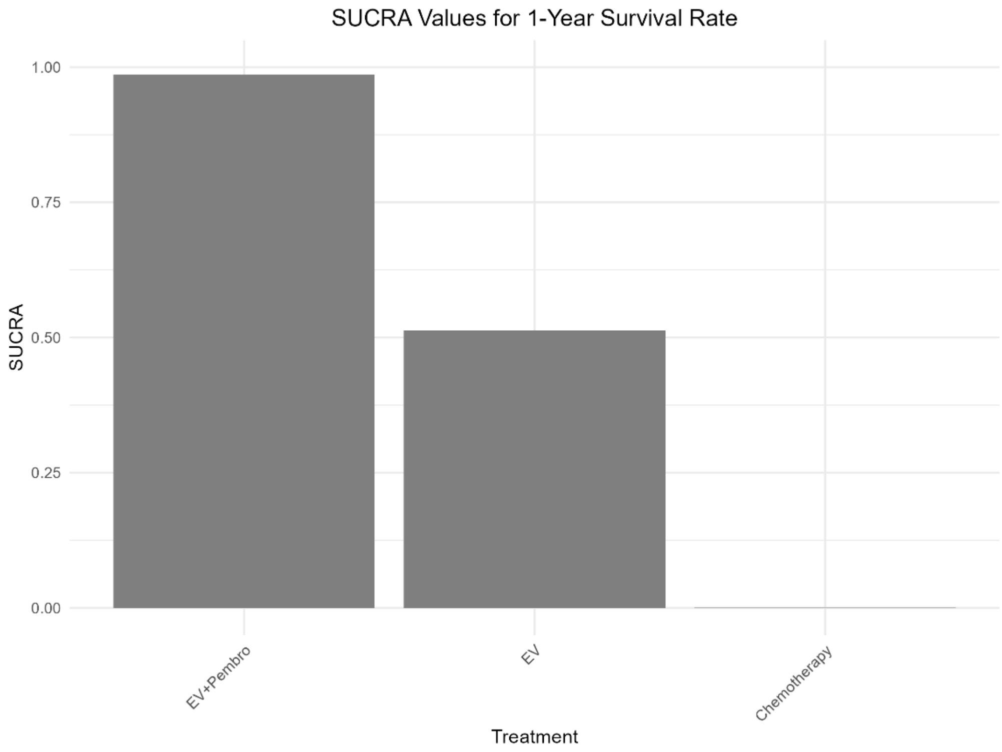

© 2025 Yajima S et al. *JAMA Network Open*

eFigure 9. Forest Plots of High-Grade AEs for mUC: Comparison of EV plus Pembrolizumab, EV monotherapy, and Chemotherapy

(A) Anemia

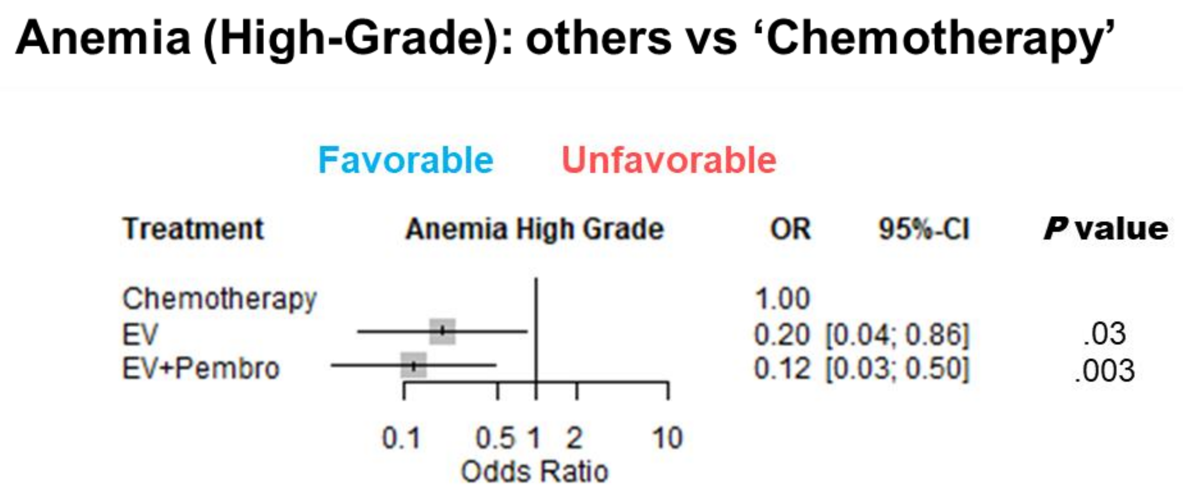

(B) Diarrhea

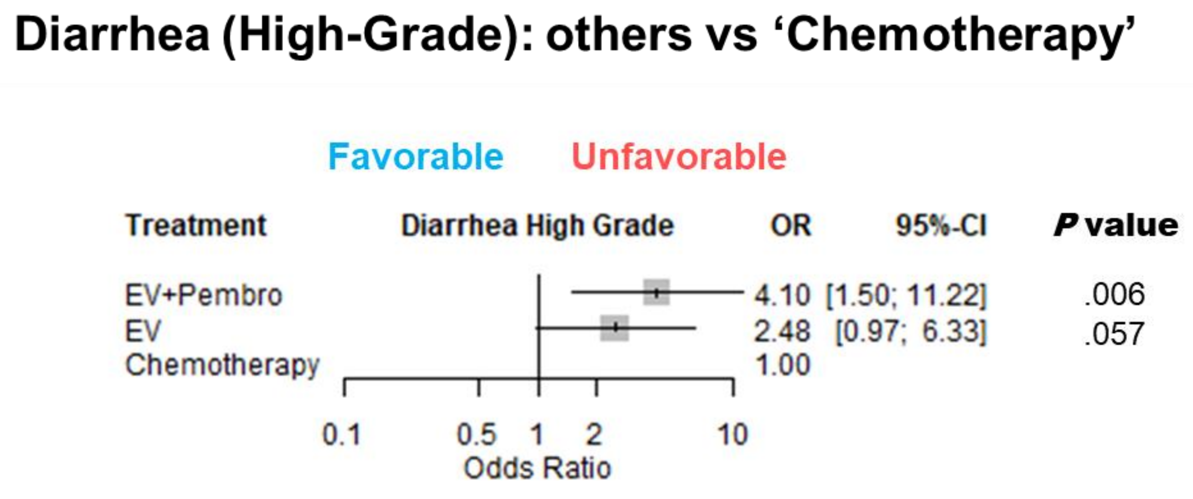

© 2025 Yajima S et al. *JAMA Network Open*

(C) Fatigue

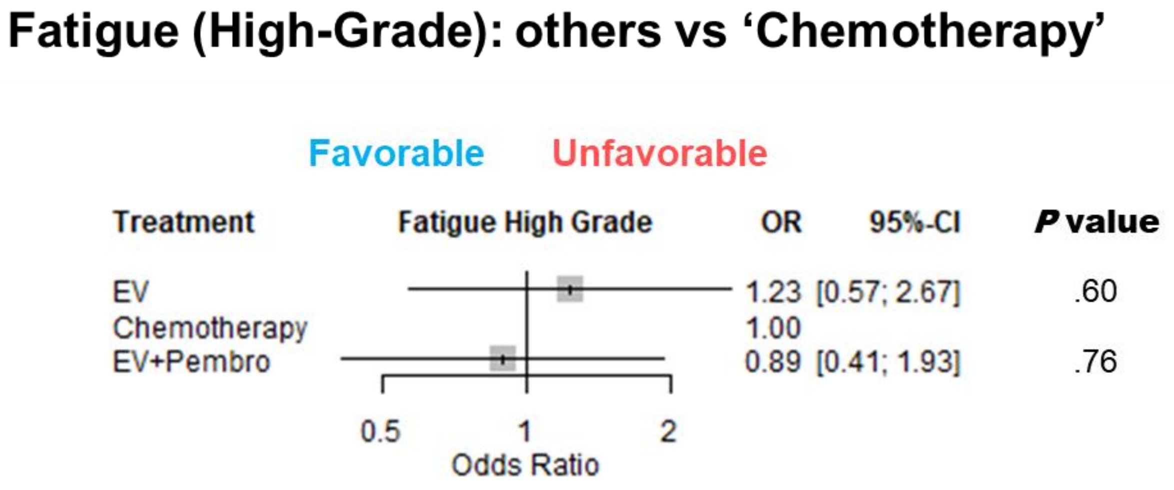

(D) Neutropenia

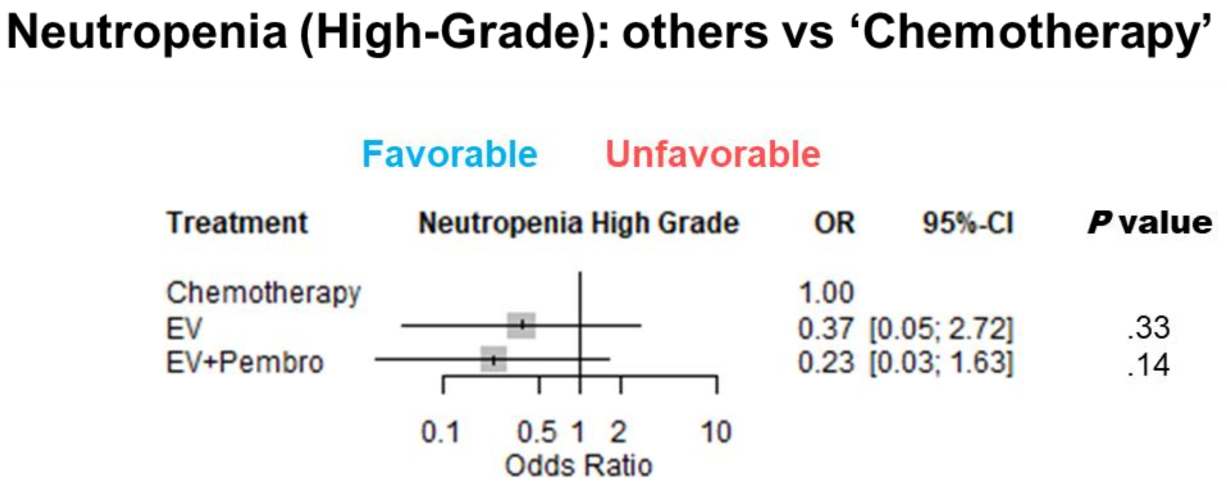

© 2025 Yajima S et al. *JAMA Network Open*

(E) Pruritus

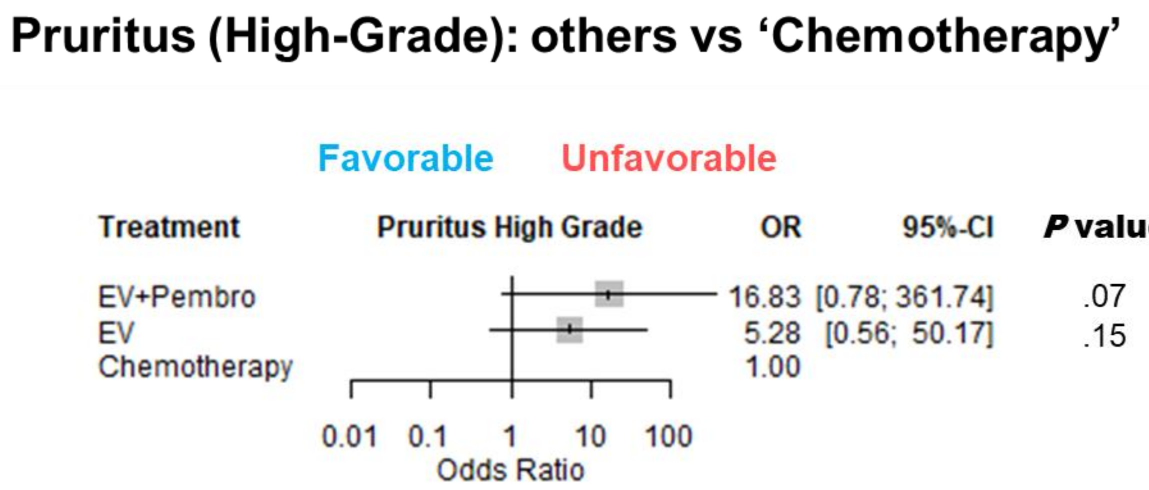

(F) Peripheral neuropathy

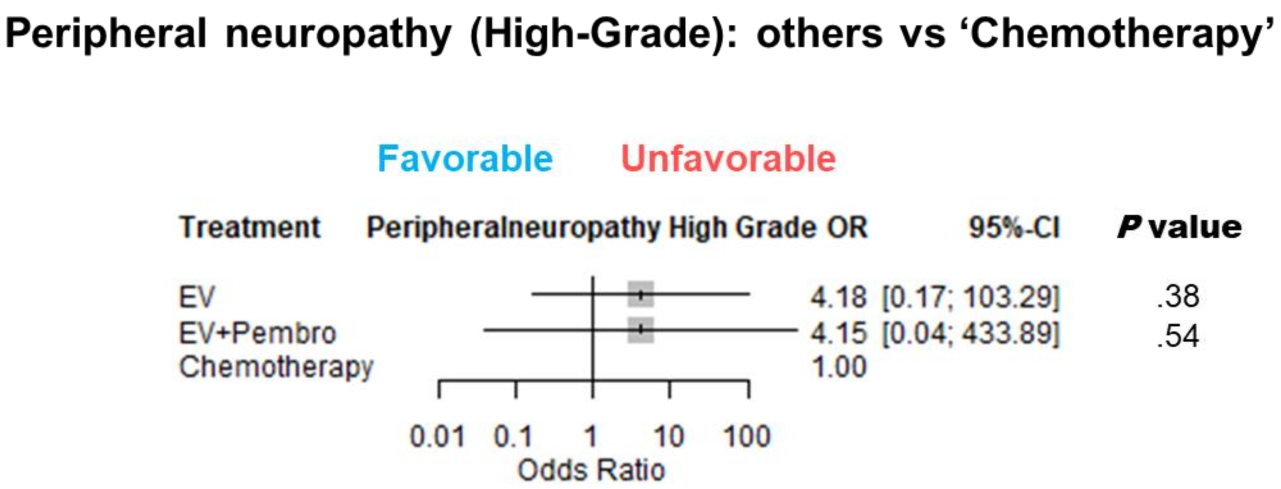

© 2025 Yajima S et al. *JAMA Network Open*

eFigure 10. SUCRA rankings of High-Grade AEs for mUC: comparison of EV plus Pembrolizumab, EV monotherapy, and Chemotherapy

(A) Anemia

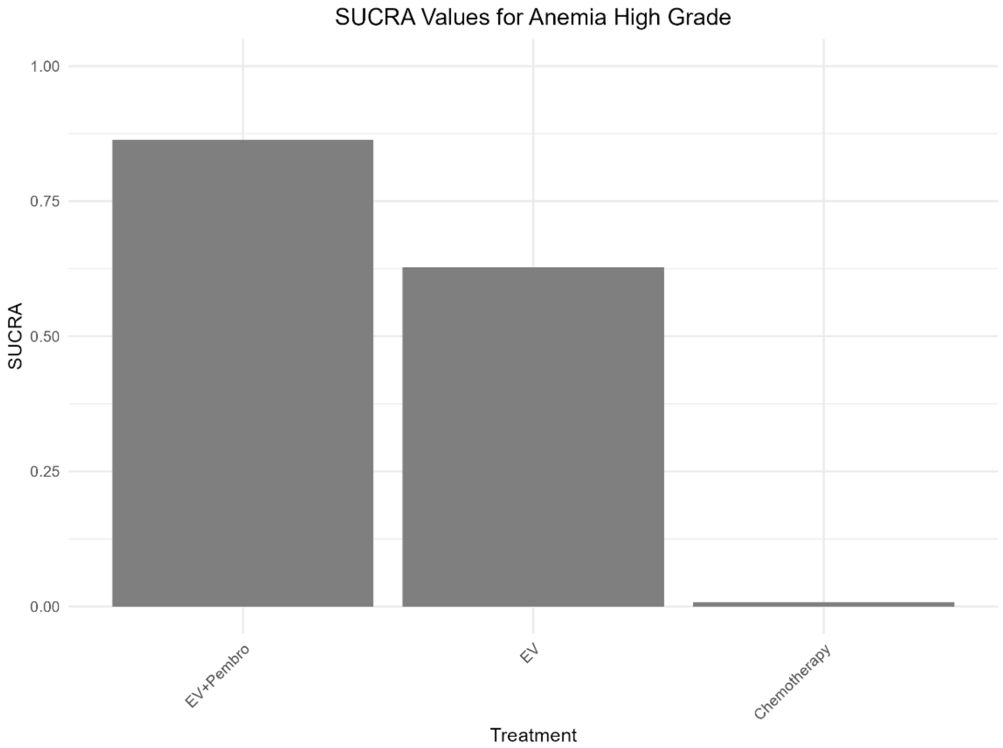

(B) Diarrhea

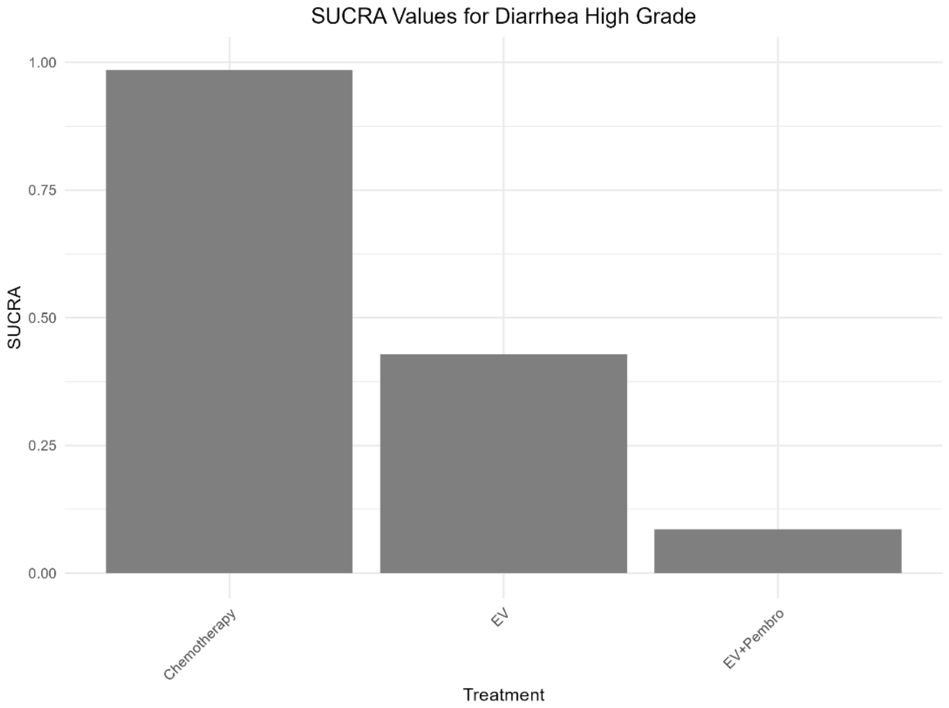

© 2025 Yajima S et al. *JAMA Network Open*

(C) Fatigue

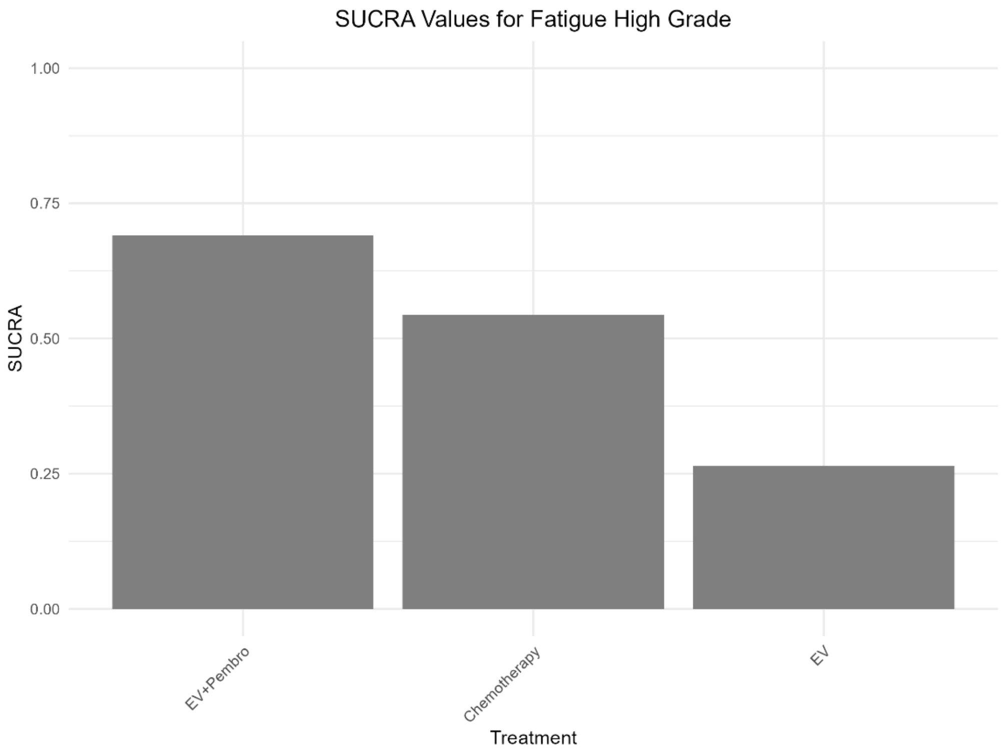

(D) Neutropenia

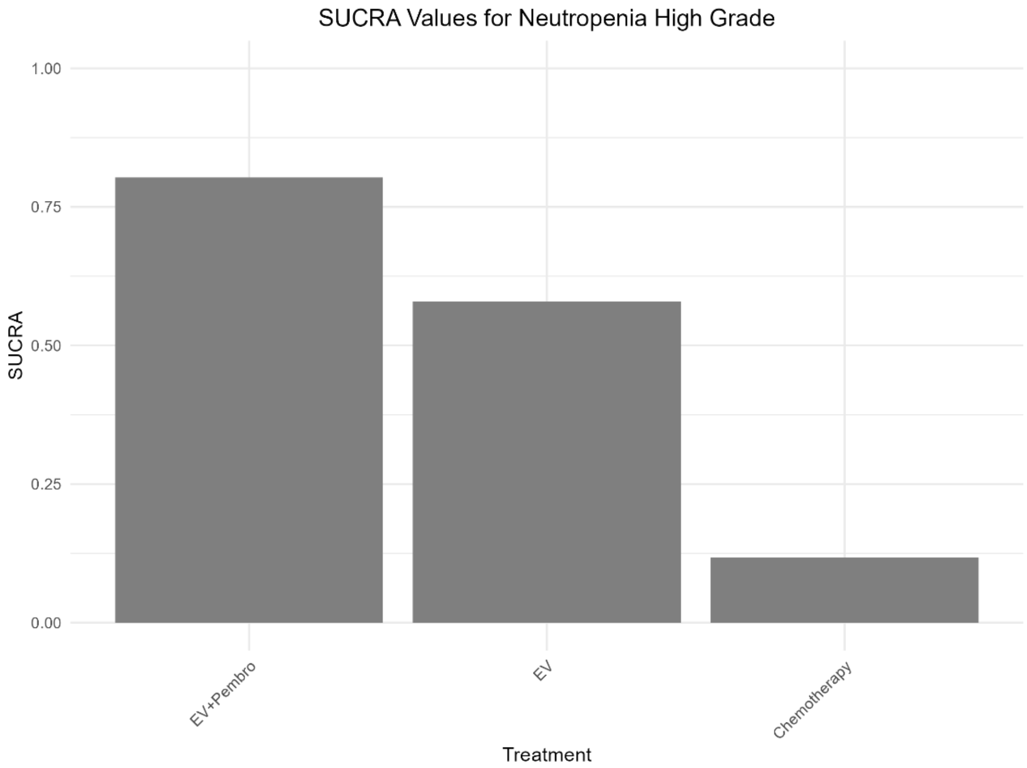

© 2025 Yajima S et al. *JAMA Network Open*

(E) Pruritus

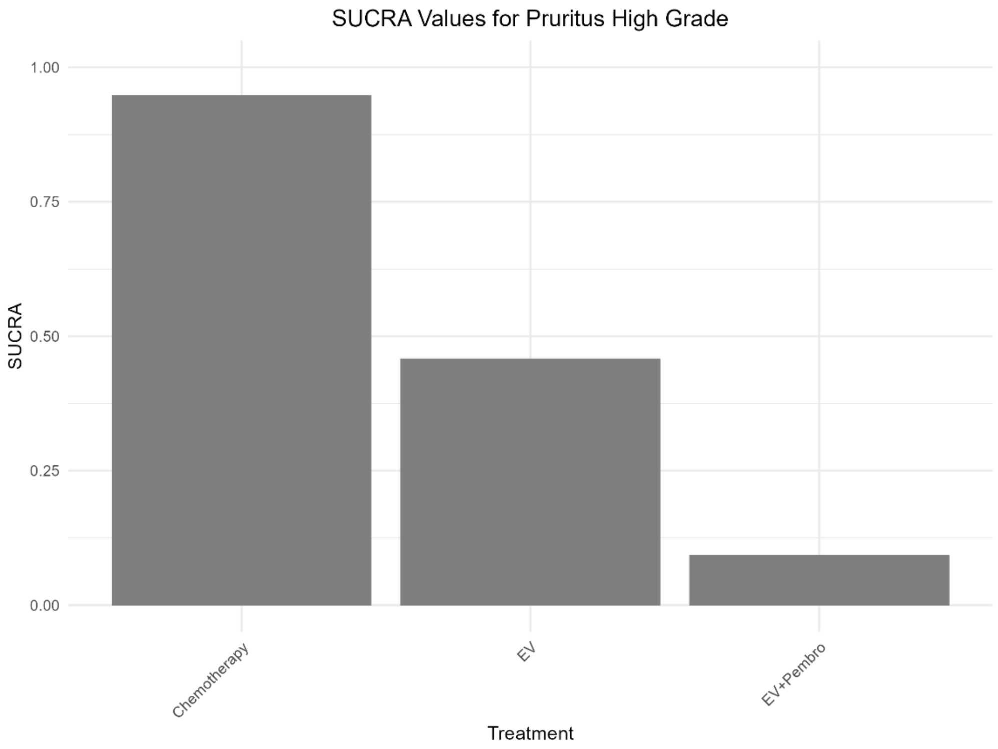

(F) Peripheral neuropathy

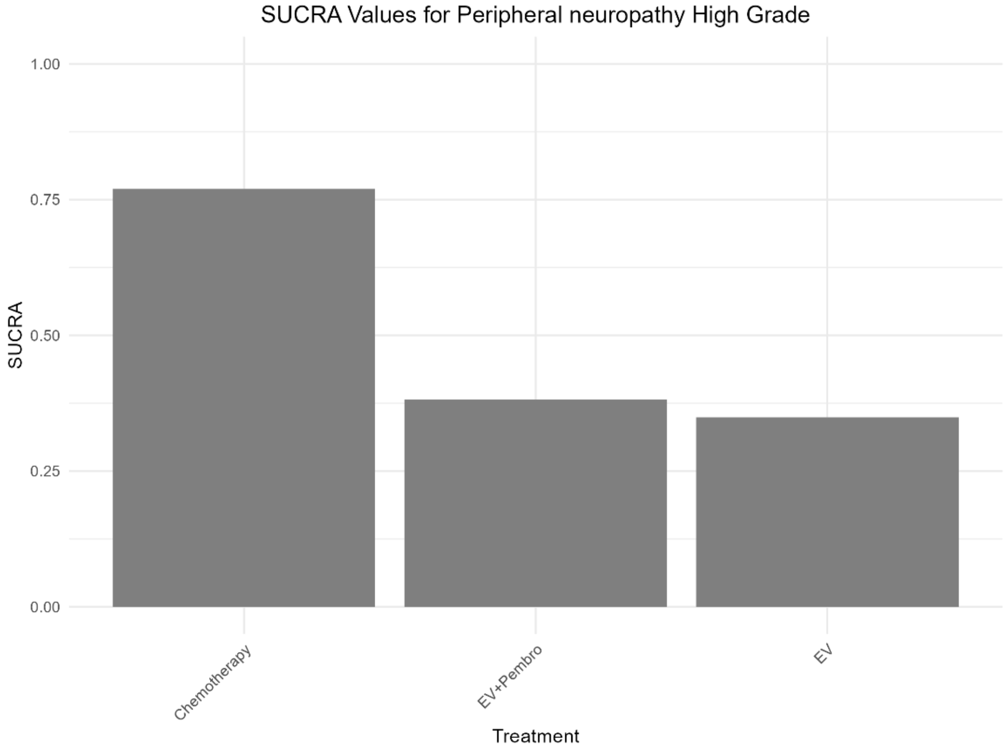

© 2025 Yajima S et al. *JAMA Network Open*

eFigure 11. Forest Plots of All-Grade AEs for mUC: Comparison of EV plus Pembrolizumab, EV monotherapy, and Chemotherapy

(A) Anemia

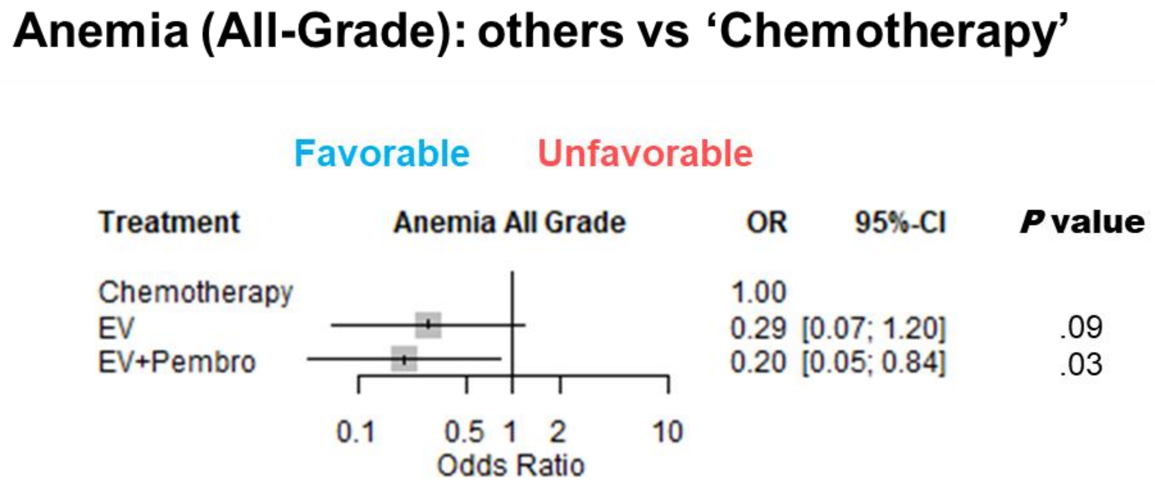

(B) Diarrhea

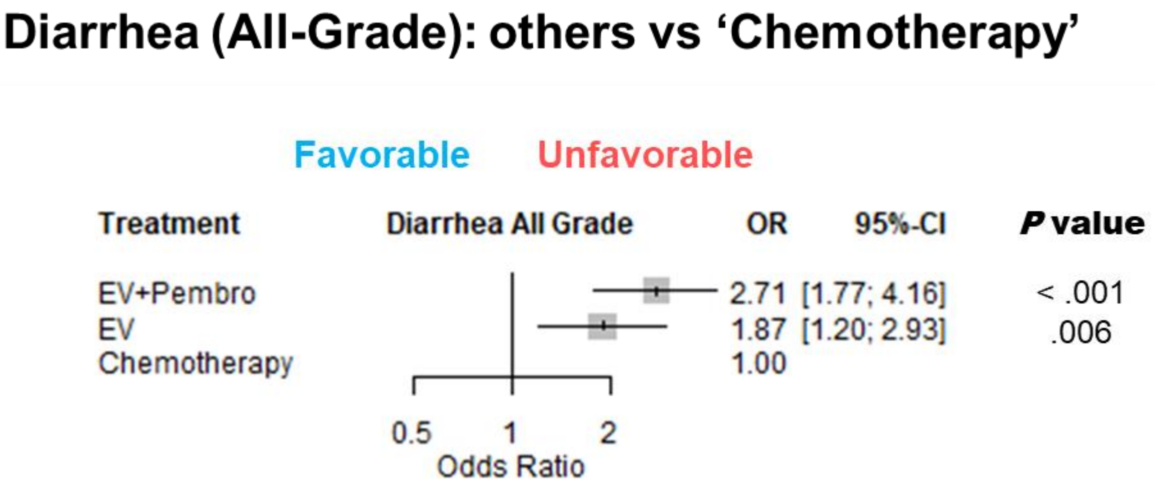

(C) Fatigue

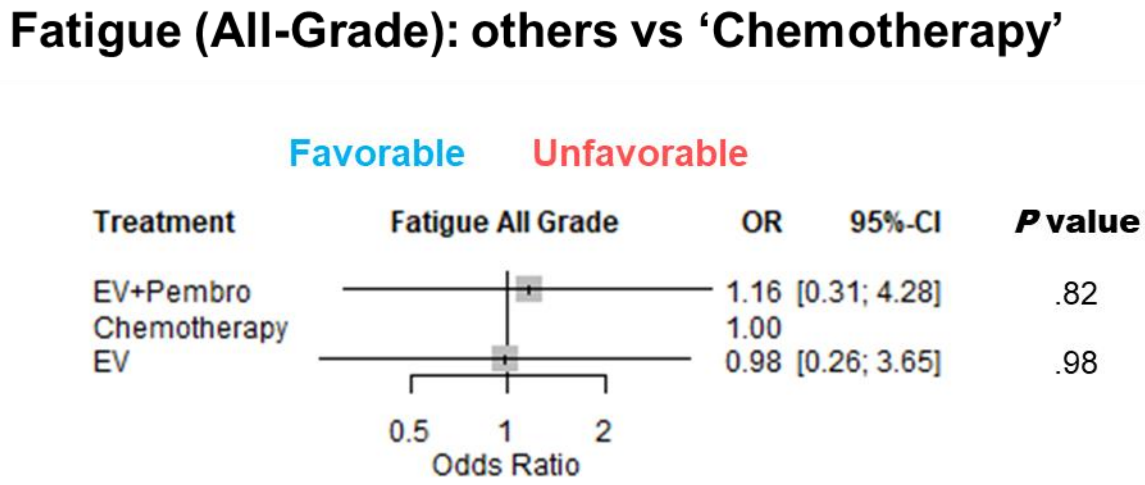

(D) Neutropenia

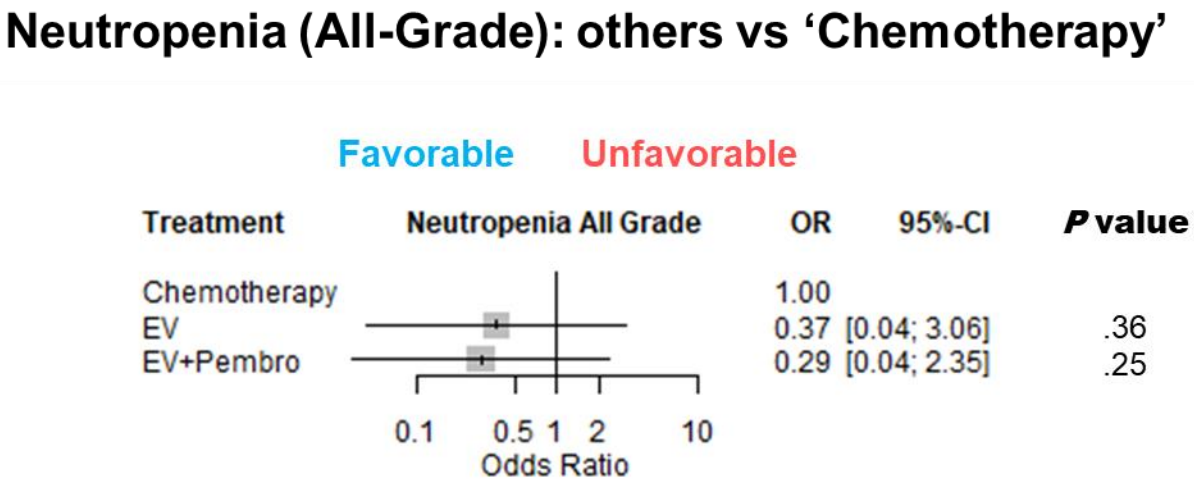

© 2025 Yajima S et al. *JAMA Network Open*

(E) Pruritus

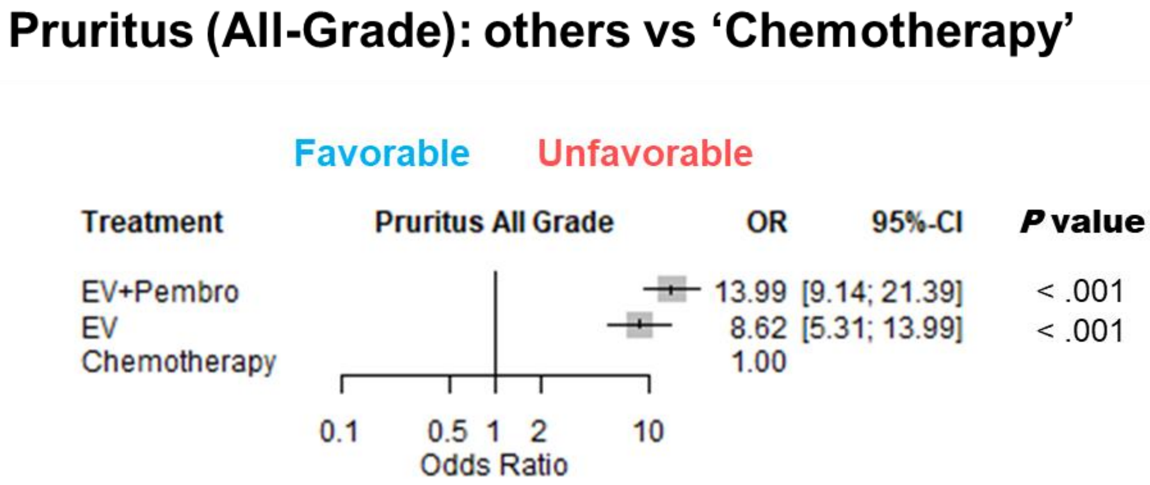

(F) Peripheral neuropathy

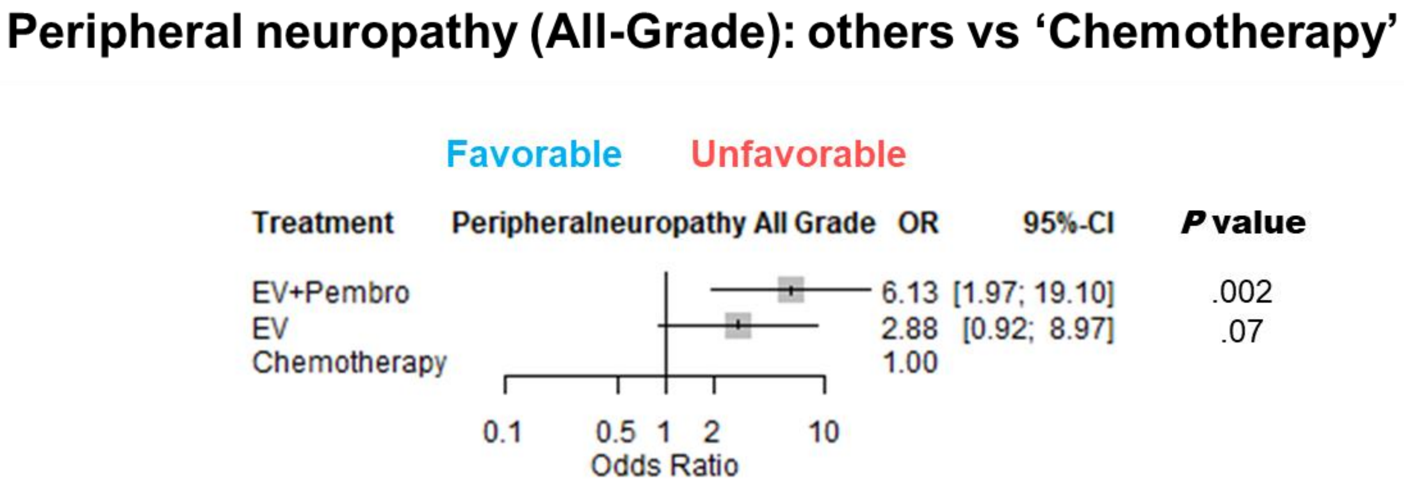

© 2025 Yajima S et al. *JAMA Network Open*
