# Supplementary material for: Enfortumab Vedotin With or Without Pembrolizumab in Metastatic Urothelial Carcinoma: A Systematic Review and Meta-Analysis
Source: JAMA Netw Open. 2025 Mar 11;8(3):e250250. doi: 10.1001/jamanetworkopen.2025.0250 (PMC11897842; doi:10.1001/jamanetworkopen.2025.0250)
Supplement: Supplement 2. — Data Sharing Statement [file jamanetwopen-e250250-s002.pdf]

Data Sharing Statement

Yajima. Enfortumab Vedotin With or Without Pembrolizumab in Metastatic Urothelial Carcinoma. *JAMA Netw Open*. Published March 11, 2025.
doi:10.1001/jamanetworkopen.2025.0250

Data
Data available: Yes
Data types: Other (please specify)
Additional Information: A list of excluded studies from the systematic review and detailed data extraction forms
How to access data: A list of excluded studies from the systematic review and detailed data extraction forms are available from the corresponding author upon reasonable request. Please contact Dr. Shugo Yajima at shuyajim@east.ncc.go.jp for access to these data.
When available: With publication

Supporting Documents
Document types: None

Additional Information
Who can access the data: N/A
Types of analyses: N/A
Mechanisms of data availability: N/A
